# Condensins Promote Chromosome Recoiling during Early Anaphase to Complete Sister Chromatid Separation

**DOI:** 10.1016/j.devcel.2010.07.013

**Published:** 2010-08-17

**Authors:** Matthew J. Renshaw, Jonathan J. Ward, Masato Kanemaki, Kayo Natsume, François J. Nédélec, Tomoyuki U. Tanaka

**Affiliations:** 1 Wellcome Trust Centre for Gene Regulation and Expression, University of Dundee, Dundee DD1 5EH, UK; 2 European Molecular Biology Laboratory, D-69117 Heidelberg, Germany; 3 Department of Biological Sciences, Graduate School of Science, Osaka University, Osaka 560-0043, Japan

**Keywords:** CELLBIO

## Abstract

Sister chromatid separation is initiated at anaphase onset by the activation of separase, which removes cohesins from chromosomes. However, it remains elusive how sister chromatid separation is completed along the entire chromosome length. Here we found that, during early anaphase in *Saccharomyces cerevisiae*, sister chromatids separate gradually from centromeres to telomeres, accompanied by regional chromosome stretching and subsequent recoiling. The stretching results from residual cohesion between sister chromatids, which prevents their immediate separation. This residual cohesion is at least partly dependent on cohesins that have escaped removal by separase at anaphase onset. Meanwhile, recoiling of a stretched chromosome region requires condensins and generates forces to remove residual cohesion. We provide evidence that condensins promote chromosome recoiling directly in vivo, which is distinct from their known function in resolving sister chromatids. Our work identifies residual sister chromatid cohesion during early anaphase and reveals condensins' roles in chromosome recoiling, which eliminates residual cohesion to complete sister chromatid separation.

## Graphical abstract

**Graphical Abstract:**

**Figure:**
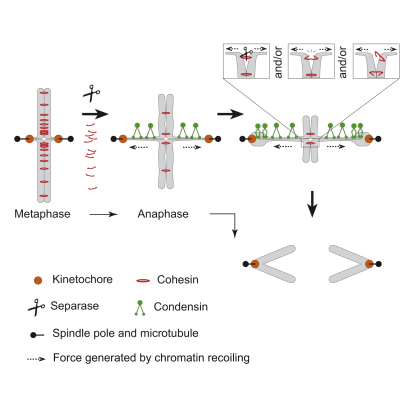

**Highlights:**

► Sister chromatid cohesion partially persists after anaphase onset in budding yeast ► Residual cohesion is partly dependent on cohesins and causes chromosome stretching ► Condensins directly promote recoiling of stretched chromosomes during early anaphase ► Chromosome recoiling generates forces to remove residual sister chromatid cohesion

## Introduction

Cohesion between sister chromatids is established during S phase and maintained until anaphase. A multisubunit protein complex cohesin plays a central role in establishment and maintenance of sister chromatid cohesion (Nasmyth, 2002). Upon anaphase onset, the cohesin component Scc1 (also called Rad21 or Mcd1) is cleaved by separase, which triggers separation of sister chromatids. Subsequently, sister chromatids are pulled toward opposite spindle poles by microtubule-dependent forces generated on the mitotic spindle. However, it is still controversial whether sister chromatid cohesion is lost completely at anaphase onset or gradually removed during anaphase (Paliulis and Nicklas, 2004). While cleavage of cohesin by separase is the trigger for sister chromatid separation at the onset of anaphase, more factors might be involved in completing this separation.

Prior to chromosome segregation triggered by removal of sister chromatid cohesion, sister chromatid DNAs must be disengaged from each other. This is facilitated by topoisomerase II, which removes catenation between sister chromatids that remains after completion of DNA replication (Wang, 2002). Decatenation of sister chromatids is a prerequisite for their resolution, which is, at least in metazoan cells, followed by their folding into compacted mitotic chromosomes (Swedlow and Hirano, 2003). A crucial regulator of these processes is condensin, a multisubunit protein complex containing two core subunits, Smc2 and Smc4, and three non-SMC subunits (Ycs4, Ycs5/Ycg1 and Brn1 in budding yeast) (Hirano, 2005; Hudson et al., 2009). While the requirement of condensins for axial chromosome compaction varies among different experimental systems, their crucial involvement in sister chromatid resolution has been identified in many organisms.

It is thought that sister chromatid resolution is achieved by cooperation between topoisomerase II and condensins (Coelho et al., 2003; Steffensen et al., 2001). Indeed, when either topoisomerase II or condensin is defective or depleted in cells, similar phenotypes are often observed. For example, during anaphase many sister chromatids fail to separate, making bridges between the two spindle poles. It is generally interpreted that these anaphase bridges are due to poor resolution and/or abnormal compaction of sister chromatids in earlier phases of mitosis (Hirano, 2005). On the other hand, it has also been suggested that condensins have additional and distinct roles during anaphase to achieve proper chromosome segregation (Wignall et al., 2003; Yanagida, 2009). The potential roles of condensins during anaphase remain elusive.

Meanwhile, several reconstitution studies have revealed that condensins can promote supercoiling of DNA, in vitro (e.g., Kimura et al., 1999). However, it is still difficult to relate in vitro supercoiling activity of condensins directly to their roles in vivo, partly because metazoan chromosomes are highly condensed in mitosis and their organization is still poorly understood.

In order to fill the gap between in vitro and in vivo studies of condensins, budding yeast *Saccharomyces cerevisiae* may prove a useful system as its chromosomes show little condensation upon the transition from interphase to mitosis (Guacci et al., 1994; see Figure S1B available online). In particular, several studies have focused on regulation of ribosomal DNA (rDNA) segregation (e.g., Freeman et al., 2000). The repetitive nature of rDNA in yeast has enabled these studies to provide insights into condensin function. In contrast to non-rDNA regions, separation of rDNA takes place in midanaphase independently of cohesins and this process requires the combined action of condensins, topoisomerase II, and other factors (D'Amours et al., 2004; Sullivan et al., 2004). On the other hand, condensins are also required for segregation of all other chromosomes that do not carry rDNA (Bhalla et al., 2002). It is still poorly understood how segregation of such chromosomes is regulated by condensins.

Here, we investigated segregation of chromosomes that do not carry rDNA, in budding yeast. Our study identifies residual sister chromatid cohesion during early anaphase and reveals condensins' roles in its elimination.

## Results

### Segregation of Sister Chromatids during Anaphase Is Accompanied by Their Regional Stretching and Subsequent Recoiling

**Figure 1:**
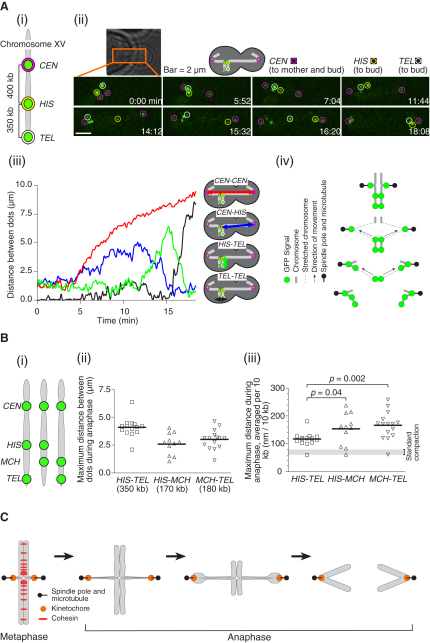
Chromosome Arms Show Regional Stretching and Subsequent Recoiling during Their Segregation (A) Observation of three loci along chromosome XV during anaphase. Cells (T4189) with *TetR-GFP* and *tetOs*, integrated at three loci as indicated in (Ai), were arrested by α factor treatment and released into fresh medium. After 80 min, GFP images were collected every 4 s for 45 min. (Aii) Representative time-lapse images showing segregation of the three GFP-labeled loci during anaphase. Pink, yellow, and white circles indicate sister *CEN*s (pulled toward opposite spindle poles), *HIS*, and *TEL* (on the sister chromatid that entered the bud), respectively. Time 0 is set arbitrarily. See Movie S1. Figure S1A shows images of other cells. (Aiii) Changes in the distances between the individual GFP-labeled loci. (Aiv) Schematic drawing of the segregating GFP-labeled loci. (B) Chromosome stretching between three different pairs of GFP-labeled loci was evaluated as in (A). (Bi) T4189, T6756, and T6876 cells carry each marked chromosome XV from left to right. (Bii) The maximum distances between the two GFP-labeled loci during their segregation in anaphase. Thick lines indicate mean values. (Biii) The maximum distances, averaged per 10 kb. Standard compaction was calculated, assuming that 10 kb spans 60–80 nm (Bressan et al., 2004; Bystricky et al., 2004). (C) Model of chromosome segregation during anaphase, accompanied by regional stretching and subsequent recoiling of a chromosome arm.

To address how sister chromatids separate along their length and segregate toward the spindle poles during anaphase, we sought to visualize multiple loci along a single chromosome arm of *S. cerevisiae*. To this end, we inserted an array of *tet* operators, which were bound by Tet repressors (TetR) fused with green fluorescent protein (GFP) and thus forming microscopic fluorescent “dots,” in the vicinity of *CEN15*, at the *HIS3* locus, and close to the telomere (called *CEN*, *HIS*, *TEL* below for simplicity) on the right arm of chromosome XV (Figure 1Ai). This chromosome arm was chosen in our study as it is relatively long (the third longest chromosome arm), and because chromosome XV does not carry any specialized chromosome regions such as rDNA or mating type loci.

**Figure 2:**
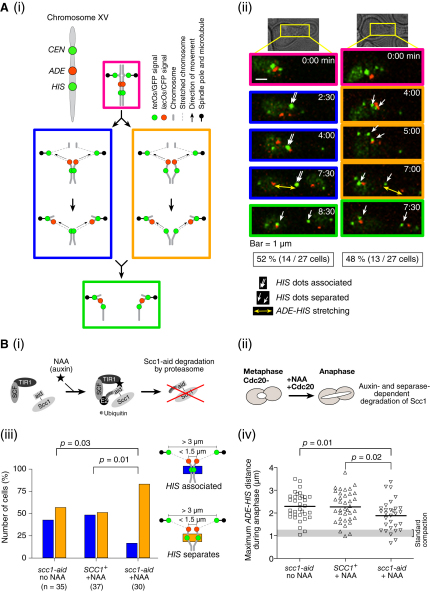
Sister Chromatid Cohesion Is Still Partially Present after Anaphase Onset and Contributes to Chromosome Stretching (A) Sister chromatids remain partially associated following the onset of anaphase. Cells (T7091) with *TetR-GFP 3×CFP-LacI tetOs lacOs* (integrated as indicated in [Ai], top) were arrested by α factor treatment and released into fresh medium. After 80 min, GFP and CFP images were collected every 30 s for 45 min. (Ai) Schematic drawing and (Aii) representative time-lapse images (time 0, start of image acquisition) showing segregation of *CEN* (*tetOs*; green), *HIS* (*tetOs*; green), and *ADE* (*lacOs*; red) loci. During the period (blue and orange rectangles) after anaphase onset (distance between sister *CEN*s >3 μm) but before *ADE* CFP dots had begun segregating toward the poles (distance between sister *ADE*s <1.5 μm), it was scored whether the sister-*HIS* GFP dots remained associated (blue rectangles) or they showed separation at least for one time point (orange rectangles). (B) Residual cohesion during early anaphase partly depends on the cohesin complex and causes chromosome arm stretching. *scc1-aid* (T8455) and *SCC1*^+^ (T8487) cells with *Pmet-CDC20 osTIR1 TetR-GFP 3×CFP-LacI tetOs lacOs* (at *CEN*, *ADE*, and *HIS* as in [Ai]) were arrested with α factor treatment, released into fresh medium, and arrested at metaphase by Cdc20 depletion for 2.5 hr. Cells were released into anaphase by re-expression of Cdc20 (by transfer to methionine drop-out medium) concomitantly with addition of NAA. Subsequently, GFP and CFP images were collected every 30 s for 45 min. T8455 cells were also treated without NAA addition. In all three conditions in (Biii), the distance between sister *CEN*s was enlarged (>3 μm) with similar timing (data not shown). (Bi) Schematic of auxin degron system. SCF: Skp1, Cullin, and F-box complex. E2: E2 ubiquitin ligase. aid: auxin-inducible degron. See Nishimura et al. (2009) for details. (Bii) Experimental procedure. (Biii) The separation of sister *HIS* GFP dots, scored as in (A). Figure S2D shows the amount of Scc1-aid protein during the time course. (Biv) The maximum distances between *ADE* and *HIS* during their segregation in anaphase of individual cells. Thick lines indicate mean values.

We tracked the motions of the three GFP dots during anaphase (Figures 1Aii and S1A and Movie S1, available online). Intriguingly, they did not move together to the bud, but moved one by one separated by distinct time intervals. We assumed that the GFP dots moved to the bud in the order of *CEN*-proximal to -distal, and this was verified by marking them with differently colored fluorescent proteins (see Figure 2A). Accordingly, each GFP dot was identified by following its position retrospectively from its position during segregation. While the distance between two *CEN*s became gradually longer (indicative of anaphase onset), the *CEN*-*HIS* distance was enlarged momentarily (Figure 1Aiii). As the *CEN-HIS* distance was subsequently shortened, the *HIS*-*TEL* distance was enlarged in turn but again only transiently. This was finally followed by segregation of sister *TEL*s.

Although the increased distance between loci along the chromatid may simply reflect unfolding of a chromosome from its organization in the mother-cell nucleus, it may also involve stretching and subsequent recoiling of a chromosome (Figure 1Aiv). Note that, in this paper, we define “stretching” as a decrease in compaction relative to a standard yeast chromosome, whose compaction is similar to a metazoan interphase chromosome (Guacci et al., 1994), whereas “condensation” is defined as an increase in compaction, when compared with a metazoan interphase chromosome.

The above result prompted us to quantify chromosome arm stretching during anaphase. Previous studies suggested that a 10 kb chromosome region normally spans about 60–80 nm (Bressan et al., 2004; Bystricky et al., 2004) and does not undergo further condensation during mitosis (Guacci et al., 1994; Figure S1B). The *HIS*-*TEL* distance was 1.7-fold longer (120 nm for 10 kb) at its maximum during anaphase compared with this resting length (Figures 1Bii and 1Biii). However, the *HIS-TEL* region may not be stretched uniformly at any given time. To test this, we marked another locus (*MCH5*; called *MCH* below) on the right arm of chromosome XV with *tet* operators (Figure 1Bi). The *HIS-MCH* and *MCH-TEL* regions, which are shorter than the *HIS*-*TEL* region, were each transiently extended by 2.1- to 2.4-fold at maximum extension (150–170 nm for 10 kb) (Figures 1Bii and 1Biii). Moreover, further shorter chromosome arm regions probably show even greater stretching for a 10 kb unit length during anaphase (Figure S1C).

The greater stretching (averaged for 10 kb) of shorter chromosome arm intervals indicates that at any given time during anaphase, stretching occurs along a relatively small chromosome arm region, rather than uniformly over a long region. We infer that, as a centromere is pulled toward a spindle pole immediately after anaphase onset, the region around the centromere is stretched (Figure 1C) and subsequent recoiling of this region is correlated with stretching of the neighboring region. In this manner, regional stretching of chromosome arms and subsequent recoiling proceed from the centromere to the telomere, until the two sister chromatids have completely separated. Stretching of a para-centromere region in anaphase has also been proposed in a previous study (Pearson et al., 2001).

### Sister Chromatid Cohesion Partially Persists after the Onset of Anaphase, which Is at Least Partly Dependent on Residual Cohesin

Chromosome stretching may indicate that sister chromatids oppose immediate separation. If so, how could this happen? One possibility is that cohesion between sister chromatids is not completely removed when anaphase is initiated. To address this, we investigated possible residual cohesion around the *HIS* locus (marked with GFP), after anaphase onset (i.e., after the distance between sister *CEN*s was enlarged) but before any spindle pulling force was applied to the *HIS* locus. To monitor this period, the *ADE2* locus (called *ADE* below) was marked with *lac* operators, bound by Lac repressors (LacI) fused with cyan fluorescent protein (CFP; Figure 2Ai, red dot). In 52% of cells sister *HIS* GFP dots remained associated (Figures 2Ai and 2Aii; blue rectangle) until after the distance between sister *ADE*s was enlarged, suggesting that sister chromatid cohesion may persist around the *HIS* locus in early anaphase before the spindle force is applied. On the other hand, in 48% of cells sister *HIS* GFP dots separated (Figures 2Ai and 2Aii, orange rectangle) before the sister *ADE* distance was enlarged, suggesting a lack of cohesion in the vicinity of the *HIS* locus after the onset of anaphase.

Nonetheless, in cells showing sister chromatid separation at the *HIS* locus, the *ADE*-*HIS* region still underwent stretching (Figure 2Aii, right, 7:00 min), and this chromosome arm stretching was due to residual cohesion (see below). In those cases, residual cohesion may still be present along the chromosome arm, but somewhere other than the vicinity of the *HIS* region (Figure 2Ai, orange rectangle). Supporting this notion, the sites of residual cohesion varied from cell to cell (Figure S2A).

If sister chromatid cohesion is still partially present during early anaphase, this may be dependent on residual cohesin that was not immediately removed by separase upon anaphase onset. If so, more efficient removal of cohesin should reduce the amount of residual cohesion in anaphase. To test this, we used an auxin-dependent degradation system (Nishimura et al., 2009), in which cohesin *SCC1* was tagged with an auxin-inducible degron (*scc1-aid*) and degraded through auxin-induced poly-ubiquitylation (Figure 2Bi) as well as through normal cleavage by separase. The *scc1-aid* strain showed growth suppression in the presence of auxin NAA (Figure S2B) and rapidly developed a defect in sister chromatid cohesion when NAA was added during metaphase (Figure S2C). We arrested the *scc1-aid* strain in metaphase by Cdc20 depletion and then added NAA concomitantly with re-expression of Cdc20 (Figures 2Bii and S2D). This increased the percentage of sister *HIS* dot separation after anaphase onset but before segregation of *ADE* loci (Figure 2Biii), suggesting that residual cohesion is at least partly dependent on cohesin.

If regional chromosome stretching occurs because residual sister chromatid cohesion opposes immediate sister separation, Scc1-aid depletion upon anaphase onset by NAA may also reduce this stretching during anaphase. This was indeed the case as the *ADE*-*HIS* region showed a reduced amount of stretching (Figure 2Biv). Collectively, we suggest that some amount of cohesin persists after anaphase onset and contributes to residual cohesion, which impedes immediate sister chromatid separation along the length of a chromosome arm, leading to regional stretching of a chromosome arm during anaphase.

### Condensin Mutants Show Inefficient or Defective Recoiling of Chromosome Arm Regions during Anaphase

How is the residual cohesion, present in early anaphase, finally removed to allow the complete separation and segregation of sister chromatids? Is this removal process regulated by a local mechanism coupled with the regional chromosome status (e.g., regional stretching or recoiling) or by a global mechanism uniformly working all along chromosome arms? Our results in Figure 1 suggest that residual cohesion must be removed, at least partly, by a local mechanism. Otherwise residual cohesion would be simultaneously removed all along chromosome arms, which would not result in the cycles of regional stretching and subsequent recoiling of small chromosome regions (see Figure 1C). The simplest hypothesis is that recoiling of regionally stretched chromosomes leads to removal of residual cohesion in the adjacent region (in the direction of the telomere).

One candidate regulator of chromosome recoiling is the condensin complex because it has DNA supercoiling activity in vitro (see Introduction). To address possible involvement of condensins in chromosome recoiling, we used two condensin mutants *smc2-8* and *ycg1-2* (Freeman et al., 2000; Lavoie et al., 2002), which showed relatively mild and severe defects, respectively, in chromosome segregation (Figure S3A).

**Figure 3:**
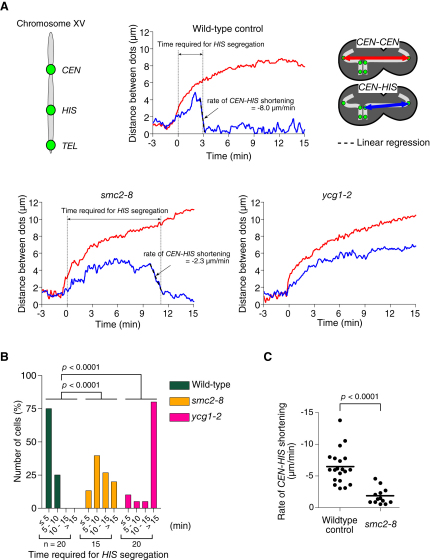
Chromosome Recoiling Is Inefficient or Defective during Anaphase in Condensin Mutants Condensin mutants (*smc2-8*; T3829 and *ycg1-2*; T3992) and wild-type control cells (T3790) with *Pgal-CDC20 TetR-GFP tetOs* (integrated at three loci as in Figure 1A) were treated with α factor at 25°C and then released into fresh medium at 35°C (restrictive temperature for *smc2-8* and *ycg1-2*) and arrested at metaphase by Cdc20 depletion for 2.5 hr. Synchronous anaphase was then induced by re-expression of Cdc20 and GFP images were acquired every 4 s for 30 min, both at 35°C. (A) GFP signals were tracked as in Figure 1A. Graphs show the *CEN-CEN* (red) and *CEN-HIS* (blue) distances (time 0: *CEN-CEN* distance reached 3 μm). Time required for *HIS* segregation is defined as the period from time 0 until the *CEN-HIS* distance became <1.8 μm in the bud. Movies S2–S4 and Figure S3B also concern these cells. (B) The percentage of cells, in which *HIS* segregation (defined as in A) completed within the indicated time window. n = number of observed cells. (C) The segregation speed of the *HIS* locus toward the bud (rate of *CEN-HIS* shortening) was measured in T3790 (wild-type control) and T3829 (*smc2-8*) cells as indicated in (A). Thick lines indicate mean values.

We compared how *CEN*, *HIS*, and *TEL* loci, marked with GFP, on chromosome XV behaved during anaphase between wild-type cells and the condensin mutants (Figures 3A, S3B, and S3C, and Movies S2–S4). In both condensin mutants, the distance between sister *CEN*s was enlarged with similar timing to wild-type, after released to anaphase (data not shown); subsequently the *CEN*-*HIS* region stretched, similarly to wild-type. However, in these mutants, there was a significant delay in the completion of *HIS* dot segregation (Figure 3B); in *smc2-8* cells, recoiling (shortening of the *CEN-HIS* region) happened with a significantly lower velocity than in wild-type cells (Figures 3A and 3C), whereas, in the majority of *ycg1-2* cells, the *CEN*-*HIS* region did not show recoiling (Figures 3A and 3B). In both condensin mutants, the *TEL* dot showed greater defects in segregation toward the bud than the *HIS* dot (see Figure S5C), consistently with a previous report (Bhalla et al., 2002). In summary, *smc2-8* and *ycg1-2* mutants showed inefficient and defective recoiling respectively, of a chromosome arm region during anaphase.

### Condensins Localize along Anaphase Chromosome Arms and Are Required during Anaphase for Their Recoiling and Segregation

What is the primary role of condensins in promoting chromosome recoiling in anaphase? Condensins may directly promote this process. Alternatively they might be required for sister chromatid separation/resolution prior to anaphase onset and, in condensin mutants, defects in this process may secondarily result in defective recoiling in anaphase.

**Figure 4:**
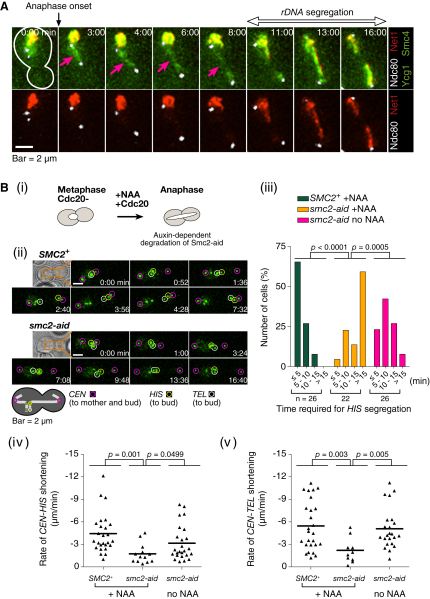
Condensins Localize on Anaphase Chromosomes and Are Required during Anaphase for Their Recoiling and Segregation (A) Condensins localize on non-rDNA chromosomal arms during anaphase. Cells (T6971) with *SMC4-3×GFP YCG1-3×GFP NET1-4×mCherry Ndc80-3×CFP* were treated as in Figure 1. After 80 min, GFP, CFP, and mCherry images were acquired every 1 min for 45 min. Representative time-lapse images are shown (0 min: start of image acquisition). The cell shape is outlined in white at 0 min. Pink arrows indicate condensins (green) localizing between kinetochore clusters (white) during anaphase before rDNA (red) segregation (bidirectional arrow). (B) *smc2-aid* (T8636) and *SMC2^+^* (T8429) cells with *osTIR1 Pmet-CDC20 TetR-GFP tetOs* (integrated at three loci as in Figure 1A) were treated with α factor, then released into fresh medium and arrested at metaphase by Cdc20 depletion for 2.5 hr. NAA was added concomitantly with Cdc20 re-expression (by transfer to methionine drop-out medium) and subsequently, GFP images were acquired every 4 s for 45 min. T8636 cells were also treated without NAA addition. In all three conditions (see Biii), the distance between sister *CEN*s was enlarged (>3 μm) with similar timing (data not shown). (Bi) Schematic of experimental procedure. (Bii) Representative time-lapse images showing the behavior of the three GFP-labeled loci during anaphase. Pink, yellow, and white circles indicate sister *CEN*s, *HIS*, and *TEL*, respectively, as in Figure 1Aii. Time 0: *CEN-CEN* distance reached 3 μm. Movies S5 and S6 concern the cells shown here. (Biii) The percentage of cells, in which *HIS* segregation (defined as in Figure 3A) completed within the indicated time window. n = number of observed cells. (Biv and Bv) The segregation speed of the *HIS* and *TEL* loci toward the bud (rate of *CEN-HIS* and *CEN-TEL* shortening), obtained by linear regression (as shown in Figures 3A and 5B), for cells that completed *HIS* or *TEL* segregation, respectively. Thick lines indicate mean values. Figure S4D shows the amount of Smc2-aid protein during the time course.

If condensins act directly on anaphase chromosomes, we may detect condensins localizing on them. To test this, we visualized the condensins Smc4 and Ycg1 together with a kinetochore component Ndc80 and an rDNA-binding protein Net1 (Figure 4A). A large amount of condensins colocalized with rDNA, as reported previously (Freeman et al., 2000). However, before rDNA showed segregation, condensin signals were found along a line between two kinetochore clusters during anaphase (Figure 4A). To confirm that these condensins are not on rDNA at the very beginning of its segregation, we prevented segregation of rDNA by removal of *CEN12* on chromosome XII harboring rDNA, using an inducible recombination system (Figure S4A). Even in this condition, condensins still localized along a line between two kinetcohore clusters in anaphase. A straightforward interpretation is that condensins localize on chromosome arms during anaphase, even if they do not contain rDNA. Similarly, condensins' localization on anaphase chromosome arms was suggested in fission yeast (Nakazawa et al., 2008).

Next, to address the role of condensin during anaphase, we inactivated condensin upon anaphase onset. We constructed a strain harboring *SMC2* with an auxin-inducible degron (*smc2-aid*), which was unable to grow (Figure S4B) and rapidly impaired localization of other condensin components on chromosomes (Figure S4C) in the presence of auxin NAA. We then arrested the *smc2-aid* strain in metaphase by Cdc20 depletion and added NAA concomitantly with re-expression of Cdc20 to deplete Smc2 (Figures 4Bi and S4D). In contrast to control cells that contained wild-type *SMC2*^+^, many *smc2-aid* cells did not show recoiling of a chromosome arm (Figures 4Bii and 4Biii and Movies S5 and S6; similarly to *ycg1-2*; see Figure 3A). In other *smc2-aid* cells, recoiling did happen, albeit with a lower velocity than in wild-type cells (Figures 4Biv and 4Bv). These defects in the *smc2-aid* cells were much greater in the presence of NAA than its absence (Figures 4Biii–4Bv). In a separate experiment, we inactivated *ycg1-2* by raising temperature after anaphase onset (during nocodazole treatment of *mad2*-deleted cells) and reached a similar conclusion (Figure S4E). These results suggest that condensins are still required during anaphase for recoiling chromosome arm regions.

### Condensins' Role in Chromosome Segregation Is Not Limited to Resolution of Sister Chromatids

**Figure 5:**
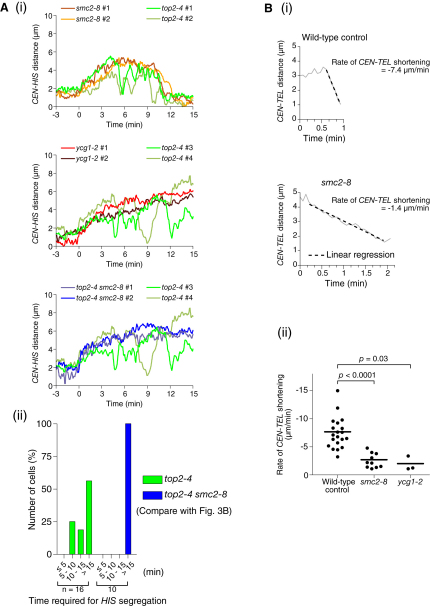
The Role of Condensins in Chromosome Segregation Is Not Limited to Resolution of Sister Chromatids (A) Condensin mutants show different behaviors of a chromosome arm locus, compared with a *top2* mutant. *smc2-8* (T3829), *ycg1-2* (T3992), *top2-4* (T3794), and *top2-4 smc2-8* (T3936) cells with *Pgal-CDC20 TetR-GFP tetOs* (integrated at three loci as in Figure 1A) were treated and analyzed as in Figure 3. (Ai) Graphs show the *CEN-HIS* distances (time 0: *CEN-CEN* distance [not shown] reached 3 μm), for two representative *top2-4* cells (#1 and 2) that completed *HIS* segregation with similar timing to the majority of *smc2-8* cells (top); and for two representative *top2-4* cells (#3 and 4) that did not complete *HIS* segregation, similarly to many *ycg1-2* cells (middle). Finally, two *top2-4* cells (#3 and 4) were compared with two representative *top2-4 smc2-8* cells (bottom). The *smc2-8* #1 and *ycg1-2* #1 cells were also analyzed in Figure 3A. Figures S3B and S5A and Movies S2–S4 and S7 also concern the cells shown here. See Figure 3A (blue line) for the change in *CEN-HIS* distance in a representative “wild-type” cell. (Aii) The percentage of cells, in which *HIS* segregation completed (*CEN*-*HIS* distance became <1.8 μm without subsequently exceeding 3 μm) within the indicated time window. Compare with the results in “wild-type,” *smc2-8*, and *ycg1-2* cells, shown in Figure 3B. n = number of observed cells. (B) The speed of telomere segregation is lower in condensin mutants. *smc2-8* (T3829), *ycg1-2* (T3992), and control wild-type cells (T3790; see their genotypes in A and Figure 3) were treated and analyzed as in Figure 3. (Bi) Changes in *CEN-TEL* distance in representative cells. Time 0 is set arbitrarily. (Bii) The speed of *TEL* segregation (shortening of *CEN-TEL* distance) toward the bud in individual cells. Thick lines indicate mean values.

The above results raised the possibility that condensins localizing along chromosome arms may directly facilitate their recoiling during anaphase. If so, condensin mutants may show different phenotypes from those of a topoisomerase II (Top2) mutant, in which chromosome segregation defects result from the inability to resolve catenation between sister chromatids (Wang, 2002) (see Figure S3A). We studied how the length of the *CEN*-*HIS* region on chromosome XV changes in the *top2-4* mutant (Holm et al., 1985) (Figures 5A, S5A, and S5B) in comparison to the condensin mutants. Some *top2-4* cells completed segregation of the *HIS* locus with similar timing to the majority of *smc2-8* cells, albeit with a delay relative to wild-type (Figures 5Ai, top, and 5Aii). Other *top2-4* cells did not complete *HIS*-locus segregation during observation, similarly to many *ycg1-2* cells (Figures 5Ai, middle, and 5Aii; Movie S7). Nonetheless, in both cases, *top2-4* cells showed vigorous back-and-forth motion of the *HIS* locus, in contrast to the condensin mutants (Figures 5Ai and S5C).

Intriguingly, such back-and-forth movements in *top2-4* cells were dependent on condensins, as they were abolished in *top2-4 smc2-8* double mutants (Figures 5Ai, bottom; 5Aii; and S5A–S5C). Furthermore, whereas the role of Top2 in chromosome segregation is largely finished by metaphase (except for the rDNA region; see Introduction), the roles of condensins are not (Figure S5D; see Figure 4B). Thus, condensin and *top2* mutants show different phenotypes. It is unlikely that this difference is simply due to allelic difference between the mutants in severity of the same defect. Thus, condensins' role in chromosome segregation is not limited to resolution of sister chromatids.

To address further the primary role of condensins in chromosome segregation, we next evaluated the segregation speed of the *TEL* GFP dot on chromosome XV. If condensin mutants are defective only in resolving sister chromatids (or directly eliminating sister chromatid cohesion), *TEL* segregation speed should be similar in wild-type and condensin mutants as sister chromatids has been separated all along their arms by this point. On the other hand, if condensins are directly involved in recoiling chromosome arms, *TEL* segregation speed of dots should be lower in condensin mutants.

We found that the speed of the *TEL* dot segregation (shortening of the *CEN*-*TEL* distance) to the bud was significantly lower in the *smc2-8* mutant (and the few *ycg1-2* cells that showed *TEL* segregation) than in wild-type cells (Figures 5Bi and 5Bii). A similar result was obtained using the *smc2-aid* mutant (see Figure 4Bv). It is unlikely that the slower *TEL* segregation speed in condensin mutants was caused by remaining sister catenation within the short region (18 kb) between the *TEL* dot and the end of the chromosome; if such catenation were involved and subsequently dissolved, the *TEL* segregation speed would have been enhanced later, but it was almost constant as it proceeded (Figure 5Bi). Collectively, these results support the notion that condensins have primary roles in recoiling chromosome arms.

### Condensins Play Active Roles in Chromosome Recoiling Independently of Sister Chromatid Resolution/Separation

**Figure 6:**
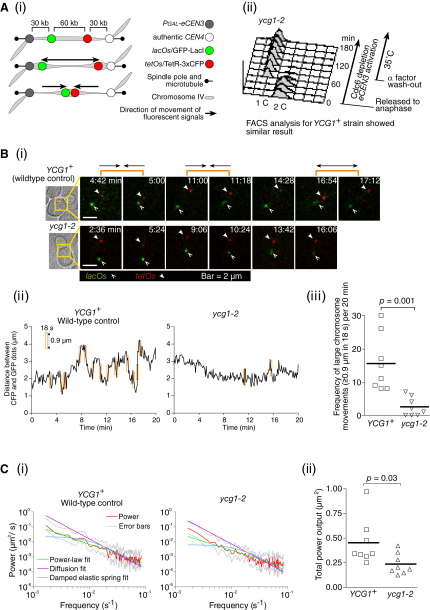
Condensins Play Active Roles in Chromosome Recoiling Independently of Sister Chromatid Resolution/Separation (A) Making an unreplicated chromosome with two centromeres. *YCG1^+^* (condensin wild-type, T5822) and *ycg1-2* (T5823) cells with *Pgal-CDC6 Pmet-CDC20 Pgal-eCEN3* (ectopic *CEN3*; integrated 120 kb to the left of the authentic *CEN4*) *TetR-3×CFP GFP-LacI lacOs tetOs* (integrated as shown in [Ai]) were arrested in metaphase by Cdc20 depletion and then released to anaphase (0 min in [Aii] FACS analyses) by re-expression of Cdc20 at 25°C. From 30 min before re-expression of Cdc20, Cdc6 expression was inhibited and e*CEN3* was activated in glucose-containing medium. Cells were then arrested by α factor treatment, followed by release (90 min in [Aii]) into fresh medium at 35°C. After 80 min, GFP and CFP images were acquired every 6 s for 20 min. (B) An unreplicated dicentric chromosome shows condensin-dependent stretching and recoiling. (Bi) Representative time-lapse images (time 0, start of image acquisition) showing *tetOs* (CFP) in red and *lacOs* (GFP) in green. Orange brackets indicate examples of stretching and recoiling of the region between the CFP and GFP dots. (Bii) Changes in the distance between the two dots in the cells shown in (Bi). (Biii) Frequency of large changes (≥0.9 μm within 18 s) in the distance between the two dots in individual cells. Thick lines indicate mean values. (C) Power spectra analyses for the oscillation of the CFP and GFP dots. (Ci) Using discrete Fourier transforms, power in the oscillation (red) was plotted as function of frequency of oscillation. Error bars (gray) show 90% confidence of the power. The power was fitted by curves, based on a motion of power-law (green), diffusion (purple), or damped elastic spring (cyan). See more detail in Supplemental Note for Power Spectrum Analyses. (Cii) Total power (variance) in individual cells. Thick lines indicate mean values.

If condensins directly promote chromosome recoiling during anaphase, we should be able to identify such condensin function even in the absence of a sister chromatid. To engineer such a situation, we inhibited DNA replication by depleting Cdc6, which is required for DNA replication initiation. We predicted that, if an unreplicated chromosome were placed under tension, it would show vigorous motion in a similar fashion to the *HIS* locus in the *top2-4* mutant (see Figure 5A). If we visualize two loci, the region between them may show stretching and shortening. To this end, we inserted an ectopic *CEN3* (e*CEN3*), under the control of a galactose-inducible promoter (Hill and Bloom, 1987), on chromosome IV (Figure 6A). Two chromosomal loci between e*CEN3* and *CEN4* were labeled with GFP and CFP. After activation of e*CEN3* by shutting off the adjacent galactose-inducible promoter, we observed the motion of the GFP- and CFP-labeled dots.

We then compared the behavior of the two dots in *YCG1*^+^ wild-type and *ycg1-2* mutant cells. In *YCG1*^+^ cells, the two dots moved vigorously after apparently coming under tension (Figure 6B). The distance between the dots was repeatedly stretched and shortened. In contrast, the movements of the two dots were less vigorous in *ycg1-2* cells (Figure 6B), although the mean distance was similar to that in *YCG1*^+^. Thus, condensins can facilitate chromosome recoiling in the absence of a sister chromatid. This result cannot be explained if condensins' exclusive role in chromosome segregation is resolving sister chromatids or directly removing sister chromatid cohesion.

The repeated oscillations of the fluorescently labeled chromosomal loci were further characterized using discrete Fourier transforms. These were used to obtain the power spectral density function (Gisiger, 2001), which shows how much power (variance) in the signal is distributed across different frequencies of the oscillations (see detail in Supplemental Note for Power Spectrum Analyses). Power spectra are useful for detecting periodic signals and for modeling dynamic processes. The power spectra of both *YCG1*^+^ and *ycg1-2* cells did not contain any periodic signal and were inconsistent with passive models of the chromosome such as a damped elastic spring (Figure 6Ci, cyan curve) or diffusion (P ∝ 1/f^2^, purple curve). The spectrum in *YCG1*^+^ was well fitted by a power-law (P ∝ 1/f^γ^; Figure 6Ci, green curve) with an exponent γ = 1.3 that is characteristic of a “flicker” noise (Gisiger, 2001). Furthermore, the magnitude of the oscillations, as quantified by the total power output, was greater in *YCG1*^+^ cells than in the *ycg1-2* mutant (Figure 6Cii). These results suggest that an active process, involving recoiling of a chromosome by condensins, is responsible for the large fluctuations of the distance between the labeled chromosomal loci.

### Condensin-Dependent Chromosome Recoiling Facilitates Elimination of Residual Sister Chromatid Cohesion

**Figure 7:**
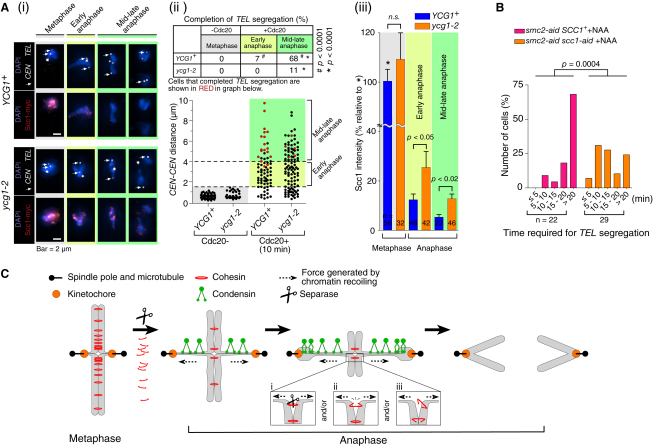
Condensin-Dependent Chromosome Recoiling Facilitates Elimination of Residual Sister Chromatid Cohesion (A) A larger amount of cohesin remains on chromosomes during anaphase when chromosome recoiling is defective. *YCG1^+^* (T7802) and *ycg1-2* (T7803) cells with *Pgal-CDC20 SCC1-18×myc TetR-GFP tetOs* (at *CEN* and *TEL* loci as in Figure 1A) were arrested at metaphase by Cdc20 depletion and subsequently released to anaphase synchronously by re-expression of Cdc20, as in Figure 3. During metaphase arrest and also 10 min after Cdc20 re-expression, chromosomes were fixed and immobilized on a slide glass immediately after cell lysis. Chromosomes were stained with DAPI and Scc1 was immunostained using an anti-myc antibody. (Ai) Representative cells. (Aii) The distance between sister *CEN* dots and the percentage of cells, in which *TEL*-dot segregation was completed (toward two distinct nuclear masses). (Aiii) The amount of Scc1, bound on chromosomes, was quantified and compared between the two strains. Bars and errors show means and SEMs, respectively. *n.s.* = not significantly different. (B) Reduction of residual cohesion restores chromosome segregation in condensin-defective cells. *smc2-aid* (T8636) and *smc2-aid scc1-aid* (T8595) cells with *osTIR1 Pmet-CDC20 TetR-GFP tetOs* (integrated at three loci as in Figure 1A) were induced to synchronous anaphase by depletion of Cdc20 followed by its re-expression, as in Figure 4B. NAA was added concomitantly with Cdc20 re-expression and after 15 min GFP images were acquired every 4 s for 45 min. The graph shows the time (after the *CEN-CEN* distance became >3 μm) when *TEL* segregation occurred to the bud. As a control, metaphase was extended for T8595 cells without re-expression of Cdc20 but with addition of NAA in the same timing; *CEN-CEN* distance did not exceed 3 μm during image acquisition (data not shown). (C) Summary for residual sister chromatid cohesion and its elimination by condensin-dependent chromosome recoiling. Separase cleaves the majority of cohesin rings at the onset of anaphase. However, due to a small amount of residual cohesins, weak sister chromatid cohesion is still present at some loci along chromosome arms, which transiently opposes sister chromatid separation and causes regional chromosome stretching. Stretched chromosome regions are recoiled by the action of condensins, leading to removal of residual cohesins, either by their cleavage facilitated by separase (Ci) or by their physical breakage/removal (Cii and Ciii) (see Discussion). Regional chromosome stretching/recoiling advances from para-centromere regions to telomeres, resulting in sister chromatid separation along the entire chromosome arms.

As discussed earlier, we hypothesized that recoiling of stretched chromosomes may lead to removal of residual cohesion between sister chromatids, as the two events proceed together along chromosome arms (see Figure 1C). To test this hypothesis, we quantified the amount of Scc1 that was bound to chromosomes, immobilized, and fixed before and after anaphase onset, in *YCG1*^+^ wild-type and *ycg1-2* mutant cells (Figure 7Ai). *CEN* and *TEL* on chromosome XV were also visualized as GFP dots (as in Figure 1A) and, based on the distance between sister *CEN*s, immobilized chromosome samples were classified as in early or mid-late anaphase (Figure 7Aii, bottom).

As expected, *TEL* dots showed segregation less frequently during anaphase in *ycg1-2* cells (Figure 7Aii, top). The amount of Scc1 on chromosomes was similar in metaphase between *YCG1*^+^ and *ycg1-2* cells (Figures 7Ai and 7Aiii). As expected, the amount of Scc1 was reduced after entry into anaphase in both cells. Intriguingly, in both early and mid-late anaphase, the amount of remaining Scc1 was significantly higher in *ycg1-2* cells (Figures 7Ai and 7Aiii). Thus, the defects in condensins led to a higher level of remaining cohesins bound to chromosomes during anaphase.

Three lines of evidence suggested that condensins facilitate removal of residual cohesins and thereby residual sister chromatid cohesion, from anaphase chromosomes indirectly by promoting their recoiling, rather than through earlier and more direct actions on cohesins. First, soon after anaphase onset but before regional chromosome stretching/recoiling reaches the *HIS* locus, *ycg1-2* cells showed similar frequencies of sister *HIS*-dot separation to *YCG1*^+^ cells (Figure S7A). Second, when treated with nocodazole, thus disrupting the spindle and therefore abolishing the effects of recoiling, *ycg1-2 mad2*Δ cells in anaphase showed similar frequency of sister *HIS*-dot separation to *YCG1*^+^ *mad2*Δ cells (Figure S7Bi). Third, the expression of a small amount of a modified Scc1, which is resistant to cleavage by separase (Uhlmann et al., 1999), led to a back-and-forth motion of the *HIS* GFP dot in anaphase (data not shown), similar to the *top2-4* mutant but distinct from condensin mutants (see Figure 5A). Thus, it is unlikely that condensins directly promote removal of cohesins from chromosomes.

If recoiling of stretched chromosomes leads to removal of residual cohesion in anaphase allowing complete sister chromatid separation, we expect that reducing residual cohesion would restore chromosome segregation in condensin-defective cells. To test this, we arrested *smc2-aid SCC1^+^* and *smc2-aid scc1-aid* cells in metaphase by Cdc20 depletion, and then added auxin NAA concomitantly with Cdc20 re-expression to degrade Smc2 and Scc1 tagged with the degron (Figure 7B). In the majority of *smc2-aid SCC1^+^* cells, the *TEL* dot did not segregate to the bud in anaphase, whereas *TEL* dot segregation occurred in the majority of *smc2-aid scc1-aid* cells. By contrast, the *TEL* dot did not segregate during an extended metaphase arrest in *smc2-aid scc1-aid* cells (data not shown), i.e., not only Scc1 degradation but also entry to anaphase was necessary for the *TEL* dot segregation. Thus reduced cohesion partially rescues chromosome segregation when condensins are defective. A corollary is that condensin-dependent chromosome recoiling facilitates elimination of residual cohesion to complete separation of sister chromatids along their arms (Figure 7C).

## Discussion

It has been a subject of debate whether sister chromatid separation is completed all along the length of chromosome arms at anaphase onset or whether this process proceeds gradually during anaphase. We found that some residual cohesion between sister chromatids is still present along chromosome arms during early anaphase of budding yeast, which temporarily opposes sister chromatid separation and causes regional chromosome stretching (Figure 7C). The residual cohesion in anaphase is at least partly dependent on cohesins, suggesting that separase cannot remove all cohesin rings (involved in cohesion) immediately at anaphase onset. Consistent with this notion, a study using micro needle manipulation in grasshopper cells showed that sister chromatid cohesion is lost gradually during anaphase (Paliulis and Nicklas, 2004).

How is the residual cohesion finally removed to complete sister chromatid separation in anaphase? We found that condensins have crucial roles in this process (Figure 7C). Condensins do not facilitate removal of residual cohesion directly, but do so indirectly by recoiling of stretched chromosomes. When a centromere is pulled toward a spindle pole immediately after anaphase onset, a para-centromere region is stretched due to residual cohesion just outside of the region. Subsequent recoiling of the para-centromere region, facilitated by condensins, leads to the removal of this residual cohesion, which is then followed by stretching of the neighboring region. In this manner, regional stretching of chromosomes and subsequent recoiling proceed from centromere to telomere, eventually leading to complete sister chromatid separation all along chromosome arms.

How does the chromosome recoiling cause removal of residual cohesion? We envisage separase-dependent and -independent mechanisms to achieve this (Figure 7C). In a separase-dependent mechanism, separase may be required for the removal of residual cohesins and chromosome recoiling may somehow help expose a cleavage site of Scc1 to separase (Figure 7Ci). We tested possible requirement of separase during anaphase for chromosome segregation by inactivating it after the onset of anaphase (Figure S7C). The result indeed suggests that separase is still required after anaphase onset for efficient chromosome segregation. Nonetheless, once anaphase is initiated, sister chromatid separation is eventually completed along a chromosome arm without the separase activity, albeit with a longer time (Figure S7C). This is in contrast to the requirement for condensins during anaphase in completing sister chromatid separation, demonstrated in a similarly designed experiment (see Figure S4E).

Thus, chromosome recoiling may also cause removal of residual cohesion in a separase-independent manner. For example, chromosome recoiling may generate forces that physically break cohesin rings that embrace sister chromatids (Haering et al., 2008) (Figure 7Cii). This is not unreasonable, given that small circular minichromosomes prematurely separate in metaphase (Tanaka et al., 1999); thus pulling forces by spindle microtubules could generate sufficient forces to break a relatively small number of cohesin rings. Alternatively, residual cohesion may be dependent on a small number of cohesin rings, which are already cleaved by separase but still loosely link the two chromatids; they may be physically removed by condensin-dependent chromosome recoiling (Figure 7Ciii). The separase-dependent or -independent mechanisms are not mutually exclusive, and all could be involved in eliminating residual cohesion.

Given the action of condensins in recoiling chromosome regions during anaphase, additional regulatory mechanisms may exist to enhance such action in anaphase. Intriguingly, yeast condensins are phosphorylated by polo-like kinase Cdc5 specifically during anaphase, which enhances the DNA supercoiling activity of condensins in vitro (St-Pierre et al., 2009); this may facilitate their action on chromosome recoiling in vivo.

Previous studies suggested that, for rDNA segregation in midanaphase, condensins and toposiomerase II must work together to resolve sister rDNAs in budding yeast (D'Amours et al., 2004; Sullivan et al., 2004). Intriguingly, the rDNA region segregates from its *CEN*-proximal to -distal part (Machin et al., 2005) and shows transient stretching (Harrison et al., 2009), which is similar to the behavior of non-rDNA regions, found in this study. However, for segregation of non-rDNA regions, condensins seem to play a direct action in recoiling stretched chromosome arm regions, rather than facilitate the function of topoisomerase II. Although we cannot exclude that condensins have additional roles in resolving sister chromatids along non-rDNA regions, we could not detect such activity in our assays (Figure S7B). We assume that, due to the highly repetitive nucleotide sequence along rDNA regions, their resolution may require further actions of topoisomerase II, which is probably assisted by condensins.

Are the roles of condensins in recoiling stretched non-rDNA regions during anaphase conserved in evolution? SMC proteins in bacteria correspond to condensins in eukaryotes, and they bind chromosomes in the vicinity of *oriC* regions in *B. subtilis* and indeed promote recoiling of chromosomes during segregation (Gruber and Errington, 2009; Sullivan et al., 2009). The SMC protein in *Escherichia coli* seems to have similar function (Danilova et al., 2007). If condensins have a similar role in chromosome recoiling in bacteria and budding yeast, this may represent an ancient mechanism to eliminate sister chromatid cohesion and complete sister chromatid separation before the evolution of cohesin cleavage by separase.

The requirement of condensins for chromosome segregation during anaphase has also been suggested in vertebrates such as the *Xenopus* egg extract system (Wignall et al., 2003), chicken DT40 cells (Vagnarelli et al., 2006), and mammalian cells (Gerlich et al., 2006). However, in these cells, condensins also play a major role in resolving sister chromatids during prophase (Coelho et al., 2003; Steffensen et al., 2001). Moreover, mitotic condensation provides stiffness to chromosomes (Marko, 2008), obscuring chromosome arm stretching during anaphase. These factors make it difficult to characterize the roles of condensins during anaphase in metazoan cells.

Nonetheless, during metaphase in metazoan cells, the centromeric chromatin comes under tension and shows dynamic motion; intriguingly, condensins are required for the structural maintenance of centromeric chromatin and also for its dynamic motion (Gerlich et al., 2006; Oliveira et al., 2005; Ribeiro et al., 2009). This condensin-dependent motion is reminiscent of the yeast chromosome behavior shown in Figure 6. Thus, when chromosome stiffness is reduced, condensins' roles in promoting chromosome recoiling may become more prominent in metazoan cells.

Our mathematical analyses of a yeast chromosome motion in vivo (see Figure 6C) have made interesting links to the condensin activity characterized in vitro. The observation that oscillations in the length of the dicentric chromosome under tension follow a “flicker” noise power spectrum provides a tentative insight into how condensin molecules could influence the global structure of the yeast chromosome. One possibility is that the system organizes spontaneously into a critical state and the oscillations are caused by “avalanches” of condensin compaction and decompaction events (Jensen, 1990). This is supported by in vitro experiments on condensin-mediated DNA compaction, which show that both DNA compaction and decompaction occur cooperatively (Cui et al., 2008; Strick et al., 2004) and that the step sizes have a long tail with the infrequent occurrence of anomalously large steps. The cooperative action between multiple condensin complexes has also been suggested by a recent structural analysis (Woo et al., 2009).

In this study, we have identified residual sister chromatid cohesion in early anaphase and its elimination mechanism, in which the cohesin and condensin complexes play crucial roles, respectively. The structural core of both complexes comprises the SMC proteins, which have been found in both prokaryotes and eukaryotes. The cohesin and condensin complexes diverged in the early evolution of eukaryotes (Cobbe and Heck, 2004), and it will be intriguing to uncover how their distinct roles in mitosis became established during evolution.

## Experimental Procedures

The background of yeast strains (W303) and methods for yeast culture were as described previously (Tanaka et al., 2007). Unless otherwise stated, cells were cultured at 25°C in YP medium containing glucose, and yeast genes were tagged at their C termini at their original gene loci by a one-step PCR method using 3×GFP (pSM1023), 4×mCherry (pT909), and 3×CFP (pT769) cassettes as PCR templates. The procedures for time-lapse fluorescence microscopy were described previously (Tanaka et al., 2007). Unless otherwise stated, time-lapse images were collected at 25°C. See more details in Supplemental Experimental Procedures.

## Supplemental Information

Document S1. Seven Figures, Supplemental Note, and Supplemental Experimental Procedures

Movie S1. Movie of the Cell Shown in Figure 1A The speed of all movies is 40 times faster than the actual movements.

Movie S2. Movie of the Cell Shown in Figure 3A (wild-type) The speed of all movies is 40 times faster than the actual movements.

Movie S3. Movie of the Cell Shown in Figure 3A (smc2-8) The speed of all movies is 40 times faster than the actual movements.

Movie S4. Movie of the Cell Shown in Figure 3A (ycg1-2) The speed of all movies is 40 times faster than the actual movements.

Movie S5. Movie of the Cell Shown in Figure 4B (SMC2+) The speed of all movies is 40 times faster than the actual movements.

Movie S6. Movie of the Cell Shown in Figure 4B (smc2-aid) The speed of all movies is 40 times faster than the actual movements.

Movie S7. Movie of Cell #3 (top2-4) Shown in Figure 5A The speed of all movies is 40 times faster than the actual movements.
