# Supplementary material for: Condensins Promote Chromosome Recoiling during Early Anaphase to Complete Sister Chromatid Separation
Source: Dev Cell. 2010 Aug 17;19(2):232–44. doi: 10.1016/j.devcel.2010.07.013 (PMC2938479; doi:10.1016/j.devcel.2010.07.013)
Supplement: Document S1. Seven Figures, Supplemental Note, and Supplemental Experimental Procedures [file mmc1.pdf]

Developmental Cell, volume *19*

Supplemental Information

Condensins Promote Chromosome Recoiling during Early Anaphase to Complete Sister Chromatid Separation

Matthew J. Renshaw,[1] Jonathan J. Ward,[2] Masato Kanemaki,[3] Kayo Natsume,[1] François J. Nédélec,[2] and Tomoyuki U. Tanaka[1,]*

[1]Wellcome Trust Centre for Gene Regulation and Expression, University of Dundee, Dundee DD1 5EH, UK
[2]European Molecular Biology Laboratory, D-69117 Heidelberg, Germany
[3]Department of Biological Sciences, Graduate School of Science, Osaka University, Osaka 560-0043, Japan
*Correspondence: t.tanaka@lifesci.dundee.ac.uk

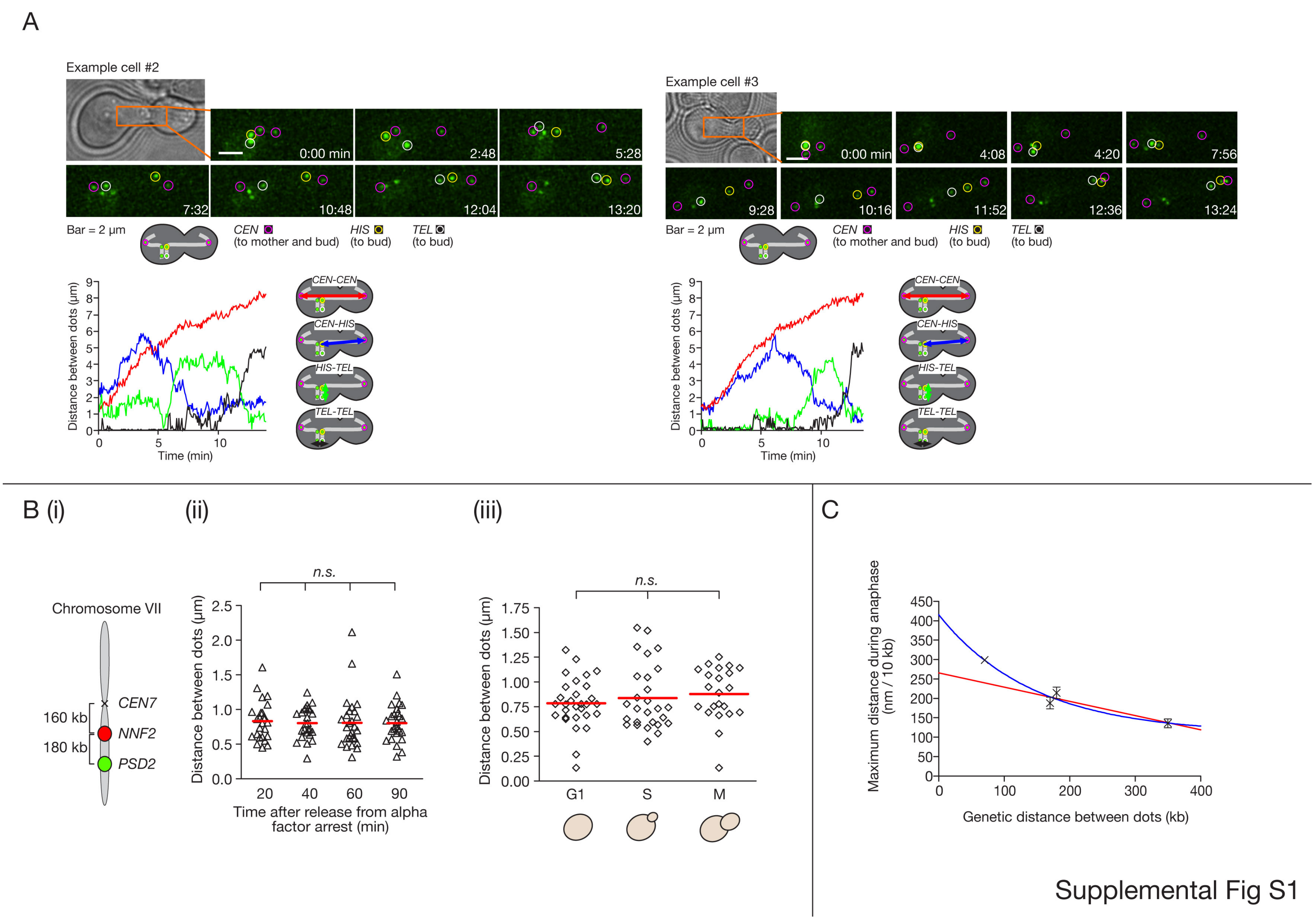

Supplemental Fig S1

Figure S1 (associated with Figure 1)

A. Observation of three loci along chromosome XV during anaphase (two other examples in addition to the cell shown in Fig 1A ii, iii).
Representative time-lapse images showing segregation of the 3 GFP-labelled loci during anaphase. T4189 (see Fig 1A) cells were treated and analyzed as in Fig 1A. In addition to the cell shown in Fig 1A (ii) (iii), two other examples (cells #2 and #3) are shown.

B. Chromosome compaction rate is similar throughout the cell cycle in budding yeast.
TetR-3xCFP GFP-LacI cells (T6605) with *tetOs* and *lacOs*, integrated along chromosome VII as shown in (i) (a gift from N. Saner, Tanaka lab), were arrested with α factor treatment and released into fresh medium. Bright field, GFP and CFP images were then acquired every 10 min for 90 min. The distance between the GFP- and CFP-labelled loci was measured in individual cells at each time point (ii) and was related to the bud size, from which cell cycle phase (G1, S and M) of the cells was estimated (iii). Cells that had entered anaphase, in which sister GFP- and CFP-dots showed separation and segregation, were excluded from the analyses. Red lines show mean values. *n.s.*= not significant.
Results. We did not observe any significant change in the distance between GFP- and CFP-labelled loci through the cell cycle. This suggests that chromosome compaction rate is similar during the cell cycle in budding yeast, at least with this temporal resolution.

C. Estimating maximum chromosome stretching over a short chromosome arm region during anaphase.
To estimate the maximum stretching of a short chromosome region during anaphase, we calculated the means of the largest stretching of *HIS-TEL*, *HIS-MCH* and *MCH-TEL* region (averaged for 10 kb; see Fig 1B) during anaphase in the top 5 cells and plotted them against the genetic distance between each pair of the two loci (350, 170 and 180 kb, respectively). The top 5 cells were selected to represent considerable stretching of the relevant chromosome regions (there was cell-to-cell variation in the extent of stretching). "×" and error bars show the mean value and standard error, respectively. Furthermore, to observe stretching of a shorter region on a chromosome arm, we used cells (T6357) with GFP- and CFP-labelled loci on chromosome IV whose interval was 70 kb (Kitamura et al., 2006).
Results. When the chromosome region was shorter, we observed less frequent stretching during anaphase, presumably because the stretching occurs very transiently. Thus, in T6357, we found chromosome stretching in only one cell during anaphase. If we consider the T6357 cell and make the data altogether fitted by a non-linear regression curve (an exponential decay; blue line; R^2 = 0.96), the extrapolated value for the 0-kb distance was 417 nm/10 kb. However, the data from only one T6357 cell is not as reliable as the other data. When the data of this one cell was not considered, the linear regression curve (red line; R^2 = 0.86) gave the extrapolated value 266 nm/10 kb for the 0-kb distance. Thus we estimate that a short chromosome arm region shows maximum stretching of 266-417 nm/10 kb during anaphase, which is 3.8-6.0 fold relative to the resting chromosome length and 55-85 % of the chromatin length with nucleosome beads but no further compaction (490 nm/10 kb).

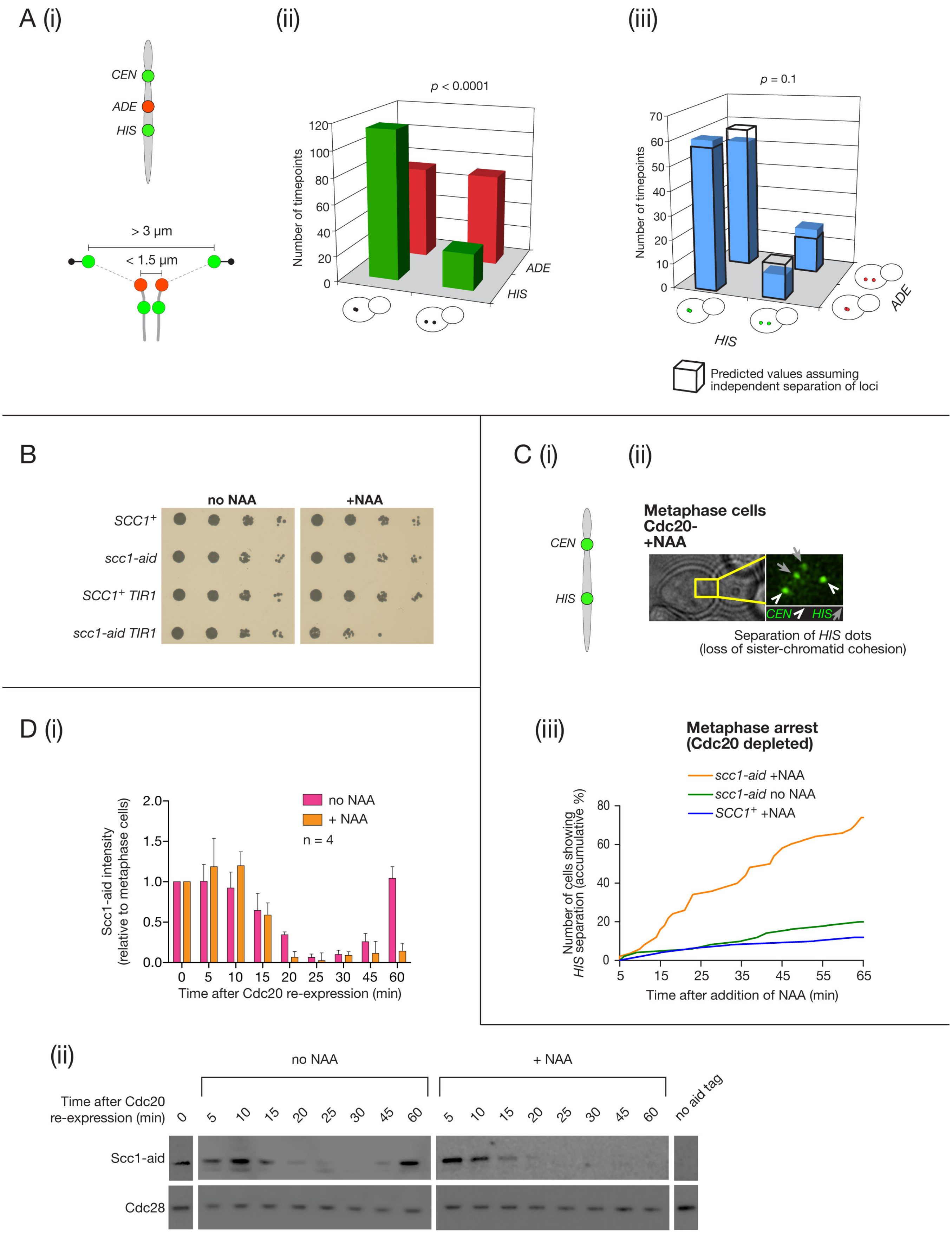

Supplemental Fig S2

Figure S2 **(associated with Figure 2)**

A. The sites of residual cohesion on chromosome arms appear to vary from cell to cell.
Fig 2A suggests that the status of residual sister chromatid cohesion at a particular locus varies from cell to cell. This variation is explained by either of the two following models (not exclusive to each other); 1) the amount of residual cohesion is higher in some cells than in others or 2) the sites of residual cohesion are different from cell to cell. To test these models, we scored frequency of separation of sister *ADE* and *HIS* dots during the short time window where cells were already in anaphase but the spindle pulling force (through stretching and recoiling in the neighbouring region in the direction of *CEN*) had not yet been applied to the *ADE* locus, using the data set obtained for Fig 2A. For this, we identified the time window (which was typically for 2-5 min) in individual cells, in which the distance between sister *CEN* dots had exceeded 3 μm (i.e. anaphase onset) but the distance between sister *ADE* dots was still less than 1.5 μm or they were associated (from this time window, we excluded 2 time points immediately before *ADE-ADE* distance exceeded 1.5 μm) as shown in (i). In (ii), we demonstrate the number of time points, in which *ADE* and *HIS* dots showed separation and association. In (iii), it is shown how separation and association of the two dots are combined at the same time points in the same cell. The wired boxes (black line) show the values of random combination, calculated from (ii).
Results. (ii) The *ADE* dots showed a higher rate of separation than the *HIS* dot, suggesting that residual cohesion may be more preferentially present along some chromosome regions than others. (iii) The above model 1) predicts that two dots would show the same status (separation or association) in individual cells, whereas the model 2) predicts that the combination of the status of the two dots would be random. The observed values (blue bars) were actually similar to the random combination, with slightly higher values for both dots being at the same status (separated or associated; although it is not statistically significant; p = 0.1). This result suggests that the sites of residual cohesion are different from cell to cell; i.e. the model 2) seems to be right. The model 1) may also contribute but only modestly.
Note (for Fig 2A and S2A). With the resolution of light microscopy, sister GFP dots, which are separated but present in very close proximity, might be discerned as “associated”. Thus it is likely that % of “associated” sister GFP dots is overestimation of cohered state of a *tetO*/*lacO* integration locus. Nevertheless, we suspect that close proximity of sister dots (such that their separation cannot be not detected in several time points before the spindle force is applied) suggests the presence of cohered sister loci in the vicinity the GFP dots. Thus, even if one particular locus shows coherence of much less than 52% (see Fig 2A ii), overall residual cohesion in a chromosome region may be strong enough, at least to temporarily oppose the spindle pulling force and cause regional chromosome stretching.

B. Growth of *scc1-aid* cells is suppressed in the presence of an auxin.
5-fold serial dilutions of *PMET-CDC20* cells with $SCC1^+$ (T8640), *scc1-aid* (T8639), $SCC1^+$ *osTIR1* (T8487) and *scc1-aid osTIR1* (T8455) were spotted onto methionine drop-out plates without NAA or containing NAA. Cells were then incubated at 25˚C for 2 days.

C. *scc1-aid* cells show a defect in sister chromatid cohesion rapidly following addition of an auxin, during metaphase arrest.
Cells (T8455) with *PMET-CDC20 osTIR1 scc1-aid TetR-GFP tetOs* (at *CEN* and *HIS* as in [i]) were arrested with α factor treatment, released into fresh medium and arrested at metaphase by Cdc20 depletion for 2.5 hours. NAA was added during metaphase arrest and images were acquired every minute for 1 hour. Control cells, with $SCC1^+$ instead of *scc1-aid* (T8487), were treated in the same way and T8455 cells were also incubated in the absence of NAA. Representative image (ii) showing separation of sister *HIS* loci. Separation of sister *HIS* loci was scored when such separation of *HIS* loci (i.e. 4 distinct GFP dots) was observed for consecutive time points (iii).

D. The change in the amount of Scc1-aid protein during anaphase.
Cells (T8455; see C) were treated as in Fig 2B. Following metaphase arrest by Cdc20 depletion, the cells were released to anaphase by re-expression of Cdc20 (transfer to

methionine drop-out medium; defined as 0 min). (i) The change in the amount of Scc1-aid (relative to metaphase-arrested cells; quantified using Western blots and calibrated using a loading control) in the presence or absence of NAA. Error bar: standard error of the mean. (ii) A representative Western blot shows the change in the Scc1-aid amount (detected using an antibody against the *aid* tag). The amount of Cdc28 was also measured as a loading control.
Results. Following re-expression of Cdc20, degradation of Scc1-aid occurred with similar kinetics in the presence and absence of NAA (although the Scc1 level may be lower from 20 min in the presence of NAA). This suggests that the auxin-dependent degradation of Scc1-aid did not precede separase-dependent Scc1 degradation, and therefore the effects of Scc1-aid degradation by auxin, shown in Fig 2B, were not due to Scc1 degradation prior to anaphase onset. Note that at 60 min (once re-budding had occurred), Scc1-aid reappeared in the absence of NAA but not in its presence, confirming the auxin-dependent degradation of Scc1-aid in this experimental setting.
Discussion. Meanwhile, Fig 2B (iii) suggests that residual cohesion decreased more quickly after Scc1-aid degradation was induced by NAA. We reason that it is difficult to clearly detect a change in residual Scc1 in early anaphase, using a Western blot in this experimental setting, because 1) separase can anyway efficiently (although not perfectly) remove most of cohesins and 2) cell-cycle synchronization is not sufficiently perfect to detect the change in small amounts of Scc1. Indeed, after re-expression of Cdc20, individual cells entered anaphase (enlargement of *CEN-CEN* distance) at various timings over an approximately 10 min period (data not shown).

Note (for Fig 2 and Fig S2): It is still possible that, in addition to residual cohesins, other factors also contribute to residual cohesion and/or regional chromosome stretching during anaphase. For example, the ORC complex may promote sister chromatid cohesion not only in metaphase (Shimada and Gasser, 2007) but also in anaphase. However, Orc2 depletion did not affect residual cohesion during early anaphase (data not shown). An alternative possibility is that the passage of chromosomes through the bud neck may generate friction leading to chromosome stretching. However, the stretching also happened for the chromatid moving to a spindle pole in the mother cell body (i.e. without passage through the bud neck), when the mother cell body was unusually large (data not shown). Thus, these factors do not seem to be substantially involved in residual cohesion or chromosome stretching in anaphase.

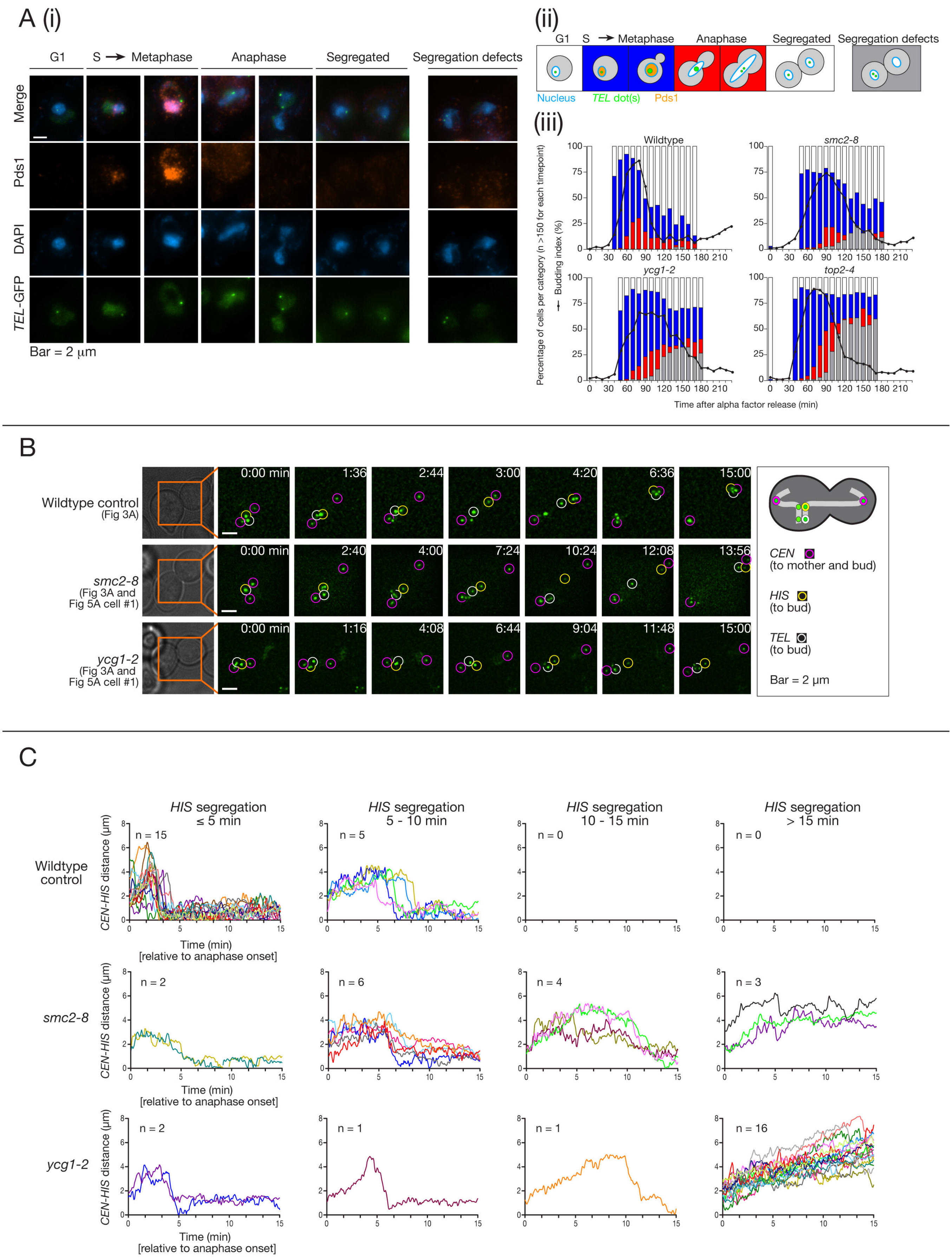

Supplemental Fig S3

Figure S3 (associated with Figure 3)

A. Chromosome segregation is frequently defective in mutants of condensins and topoisomerase II.
Condensin mutant (*smc2-8*: T4778 and *ycg1-2*: T4815), *top2* mutant (*top2-4*: T4816) and wild-type control (T4777) cells with *TetR-GFP Pds1-18myc TEL-tetOs* (on chromosome XV as indicated in Fig 1A i) were treated with α factor at 25 ˚C and subsequently released into fresh medium at 35 ˚C. All strains harboured Pds1 (securin in budding yeast), which is tagged with *myc* epitopes, to evaluate cell-cycle stages. They also had GFP-labeled *TEL* to evaluate the status of chromosome segregation. To prevent cells from entering the next cell cycle, α factor was added again when >50 % cells showed bud emergence. Aliquots of cells were fixed every 10 min after >15 % cells had shown bud emergence. Nuclei were subsequently stained with DAPI and the *myc* tag was immuno-stained using an anti-myc antibody. Cells in S phase and in metaphase were discerned by the presence of Pds1 in nuclei (blue box and bar) while cells in anaphase were recognized as those with elongated nuclei containing no Pds1 signals (red box and bar). Finally, in cells with separated nuclei, proper (white box and bar) and defective (gray box and bar) chromosome segregation was scored, based on distribution of the GFP-labeled *TEL* locus into the two nuclei. (i) Representative images, (ii) Schematic drawing of a cell in each phase and category, (iii) Percentage of cells that belong to each phase and category.

B. Time-lapse images of the wild-type, *smc2-8* and *ycg1-2* shown in Fig 3A.
Representative time-lapse images of chromosome XV segregation during anaphase in the wild-type control (T3790), *smc2-8* (T3829; cell #1) and *ycg1-2* (T3992; cell #1) cells, which are shown in Fig 3A. Pink, yellow and white circles indicate sister *CEN*s (pulled towards opposite spindle poles), *HIS* and *TEL* (on the sister chromatid that entered the bud), respectively. See Fig 3A for changes in *CEN-CEN* and *CEN-HIS* distances in these cells. See also Movies 2-4.

C. Changes in *CEN-HIS* distance in wild-type control, *smc2-8* and *ycg1-2* cells, shown in Fig 3B (more examples in addition to the cells shown in Fig 3A).
Wild-type control, *smc2-8* and *ycg1-2* cells (T3790, T3829 and T3992: see Fig 3) were treated and analyzed as in Fig 3A. Changes in *CEN-HIS* distance in multiple cells are shown in overlay with colours. Cells were grouped as in Fig 3B depending on the time required for *HIS* segregation (as defined in Fig 3A). Time 0 was defined as the time when the distance between sister *CEN*s (*CEN-CEN* distance; not shown) reached 3 μm.

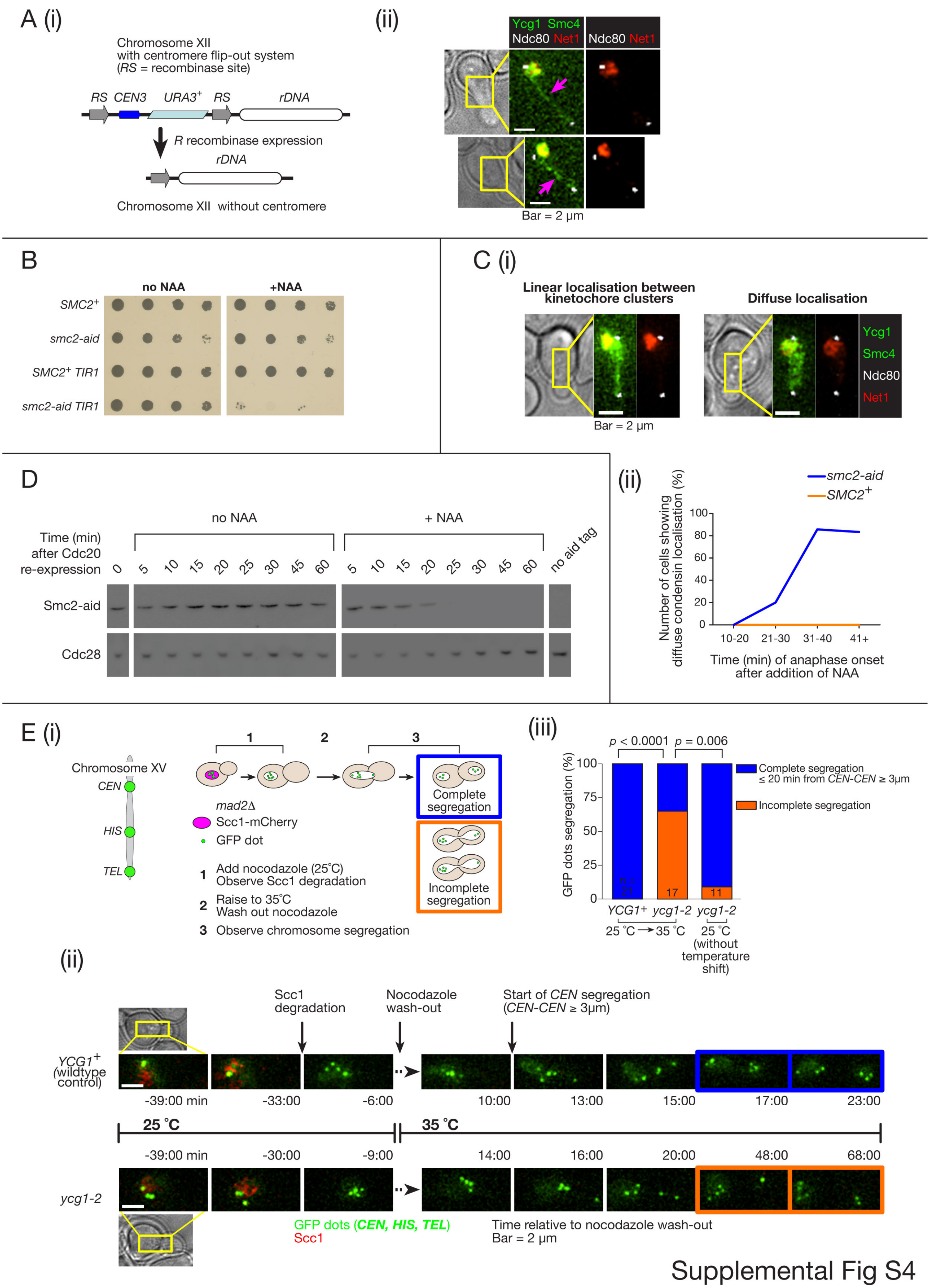

Supplemental Fig S4

Figure S4 (associated with Figure 4)

A. Removal of the centromere on chromosome XII confirms that condensins localize on non-rDNA chromosome arms during anaphase.
Cells (T7053), similar to T6971 (see Fig 4A) but also containing *cen12Δ::RS-CEN3-RS* (*RS*, recombination site) and *PGAL-R* (*R*; *Z. rouxii* recombinase) were arrested by α factor treatment and *R* expression was induced. Cells were then released into fresh medium and images were acquired as in Fig 4A. Following kinetochore segregation, no cells (out of 59) showed rDNA segregation during observation (>20-30 min after the onset of anaphase). The lack of rDNA segregation and a flip out of *CEN12* (confirmed by PCR) were dependent on expression of *R*. (i) Schematic drawing of the centromere flip-out system integrated into chromosome XII. (ii) Representative images showing condensin localization between kinetochores (pink arrows) in the absence of rDNA segregation.
Results. Fig 4A suggested that before rDNA showed segregation, condensin signals were found along a line between the two kinetochore clusters during anaphase. To confirm that these condensins are not on rDNA at the very beginning of its segregation, we replaced *CEN12* (on chromosome XII harbouring rDNA) with *CEN3* flanked by recombination sites of *Z. rouxii* R recombinase (Matsuzaki et al., 1990). The centromere on chromosome XII was then removed by expressing the *R* recombinase, which prevented rDNA segregation in all (n=59) anaphase cells that we observed. Even in these conditions, condensins still localized along a line between two kinetcohore clusters in anaphase.

B. *smc2-aid* cells cannot grow in the presence of an auxin.
5-fold serial dilutions of *PMET-CDC20* cells with *SMC2*$^+$ (T8640), *smc2-aid* (T8641), *SMC2*$^+$ *osTIR1* (T8429) and *smc2-aid osTIR1* (T8636) were spotted onto methionine drop-out plates without NAA or containing NAA. Cells were then incubated at 25˚C for 2 days.

C. *smc2-aid* cells show a reduced level of other condensin components on anaphase chromosomes following addition of an auxin.
Cells (T8593), similar to T6971 (see Fig 4A) but also containing *smc2-aid* and *osTIR1,* were arrested by α factor treatment. Cells were then released into fresh medium. After 70 min NAA was added and images were acquired as in Fig 4A. As controls, cells with *SMC2*$^+$ instead of *smc2-aid* (T8594) were treated in the same way and T8593 were also treated without NAA. (i) Representative images showing linear (left) and diffuse (right) localization of condensins (green) between kinetochore clusters (white) during anaphase, before rDNA (Net1: red) segregation. (ii) Cells were categorized based on their timing of anaphase onset (as the distance between two kinetochore clusters exceeded 3 μm) relative to addition of NAA and the condensins' localization pattern was scored (n = 4-10 cells per group). T8593 cells without NAA showed linear localization of condensins during anaphase (n = 9; data not shown).

D. The change in the amount of Smc2-aid protein during anaphase.
Cells (T8636; see B) were treated as in Fig 4B. Following metaphase arrest by Cdc20 depletion, the cells were released to anaphase by re-expression of Cdc20 (transfer to methionine drop-out medium; defined as 0 min) in the presence or absence of NAA. A representative Western blot shows the change in the amount of Smc2-aid (detected using an antibody against the *aid* tag). Cdc28 was also detected as a loading control.
Results. The auxin-dependent degradation of Smc2-aid occurred during 10-25 min; this timing was similar to that of separase-dependent Scc1 degradation (see Fig S2D, no NAA). This confirms that the effects of Smc2 degradation with auxin, found in Fig 4B, were not due to condensin degradation prior to anaphase onset.

E. Condensins are still required after anaphase onset for chromosome recoiling and segregation.
ycg1-2 (T7163) and *YCG1*$^+$ wild-type (T7162) cells with *mad2Δ SCC1-4×mCherry TetR-GFP tetOs* (integrated at three loci as in Fig 1A) were treated with nocodazole at 25 °C, both of which

entered anaphase with their mitotic spindle disrupted (i, step 1). The temperature of the cell culture was then shifted to 35 °C, nocodazole was washed out (defined as time 0 in [ii]) and cells were incubated in fresh medium (still at 35 °C) to allow cells to form a bipolar spindle (i, step 2). In 14.4 % (21/146) of *YCG1*$^{+}$ cells and in 13.1 % (17/130) of *ycg1-2* cells (difference is not significant), sister *CEN15*s showed segregation to the opposite spindle poles while in the other cells both sisters segregated to one pole. We focused on cells in which Scc1 was reduced in their nuclei (i.e. cells entered anaphase) before the temperature shift and sister *CEN15*s segregated to opposite poles. We then followed how *HIS*- and *TEL*-GFP dots segregated to each nucleus (i, step 3). (ii) Images of representative cells. (iii) The percentage of cells in which all three dots did (blue) or did not (orange) complete segregation (to each cell body) within 20 min after *CEN*-*CEN* distance exceeded 3 μm. In the majority of *ycg1-2* cells, no *TEL* segregation occurred within 40 min or longer after *CEN* segregation. *n*= number of analyzed cells.

Results. We sought to inactivate the *ycg1-2* mutant by shifting to a restrictive temperature (35 °C) after anaphase onset (i). To this end, we treated *mad2*-deleted cells with nocodazole to depolymerise microtubules. As a result of their defect in the spindle assembly checkpoint (Musacchio and Salmon, 2007), these cells entered anaphase, as confirmed by the decrease of Scc1 in the nucleus. We then shifted the culture temperature to 35 °C and washed out nocodazole, allowing cells to form a spindle and initiate chromosome segregation. In contrast to a wild-type *YCG1*$^{+}$ control, the majority of *ycg1-2* cells did not show recoiling of the *CEN-HIS* region after the temperature shift, leading to failure in completing chromosome segregation (ii and iii). Without the temperature shift, only a few *ycg1-2* cells showed such a defect, confirming that the phenotype observed was indeed due to the inactivation of condensins during anaphase. We conclude that condensins are still required during anaphase for recoiling chromosome arm regions.

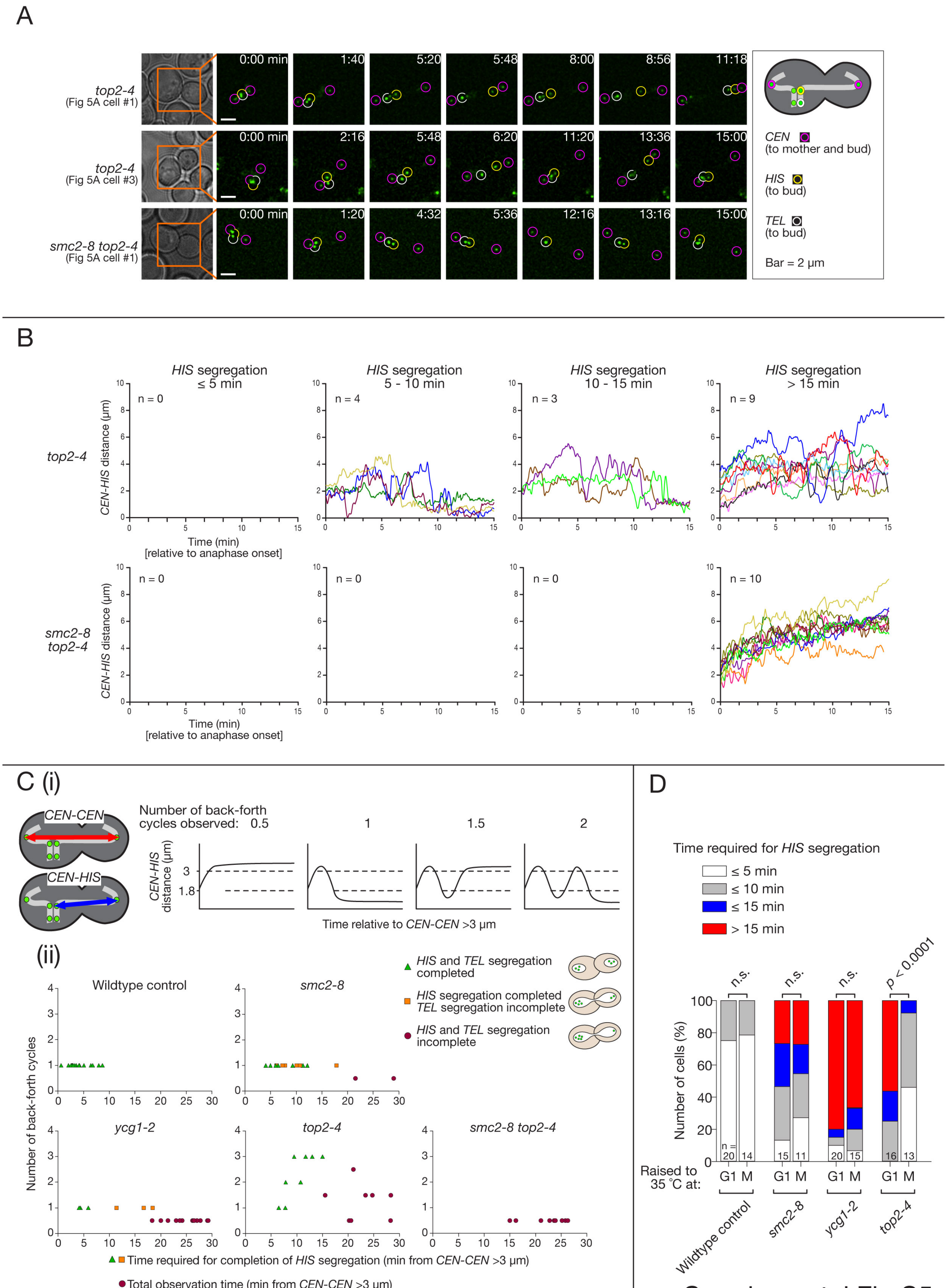

Supplemental Fig S5

Figure S5 (associated with Figure 5)

A. Time-lapse images of *top2-4* and *smc2-8 top2-4* cells shown in Fig 5A.
Representative time-lapse images of chromosome XV segregation during anaphase in *top2-4* (T3794; cells #1 and 3) and *smc2-8 top2-4* (T3936; cell #1) cells, which are shown in Fig 5A. Pink, yellow and white circles indicate sister *CEN*s (pulled towards opposite spindle poles), *HIS* and *TEL* (on the sister chromatid that entered the bud), respectively. See Fig 5A for changes in *CEN-HIS* distance in these cells. See also Movie 7. Compare with the cells shown in Fig S3B.

B. Changes in *CEN-HIS* distance in *top2-4* and *top2-4 smc2-8* cells, shown in Fig 5A ii (more examples in addition to Fig 5A i).
top2-4 and *top2-4 smc2-8* cells (T3794 and T3936: see Fig 5) were treated and analyzed as in Fig 5A. Changes in *CEN-HIS* distance in multiple cells are shown in overlay with colours. Cells were categorized as in Fig 5A ii, based on the time required for *HIS* segregation (as defined in Fig 3A). Time 0 was defined as the time when the distance between sister *CEN*s (*CEN-CEN* distance; not shown) reached 3 μm.

C. *top2-4* mutant shows vigorous back-and-forth motions of a chromosome arm locus during anaphase, in contrast to condensin mutants.
The behaviour of the *HIS* locus in *ycg1-2* (T3992), *smc2-8* (T3829), *top2-4* (T3794), *smc2-8 top2-4* (T3936) and wild-type control (T3790) cells, whose genotypes are shown in Fig 3 and 5A, was analyzed using the data sets acquired for Fig 3 and 5A (GFP images were acquired every 4 sec for 30 min). (i) Diagrams of 'cycles' defined by the change in *CEN-HIS* distance. Back-and-forth motions of the *HIS* locus were scored by counting the number of 'cycles' during observation after the *CEN-CEN* distance had exceeded 3 μm (time 0). A complete cycle was defined as *CEN-HIS* distance elongating to > 3 μm followed by shortening to <1.8 μm. Half cycles indicate that the *HIS* dot ended up in the mother cell at the end of observation. (ii) In individual cells, the time required for final *HIS* segregation (entry to the bud without subsequent travels to the mother cell) is shown in correlation with the number of cycles (defined in [i]), which were observed by completion of *HIS* segregation (green triangles, orange squares). In cells where the *HIS* locus did not complete segregation during image acquisition, the observation time (from *CEN-CEN* distance exceeding 3 μm) is shown in correlation with the number of cycles, counted by the end of observation.
Results. top2-4 cells showed an increase (1.5 or more) in the back-and-forth cycles when there was a delay in *HIS* segregation. In contrast, condensin mutants did not show such back-and-forth cycles, even when there was a delay in *HIS* segregation. The results suggest that the presence and absence of the back-and-forth motion of the *HIS* locus is not due to allelic difference between the *top2* and condensin mutants in severity of the same defect. Moreover, the back-and-forth motion of the *HIS* locus in *top2-4* is dependent on condensins as it was abolished in *smc2-8 top2-4* cells.

D. Top2 can largely finish its roles in chromosome segregation (for non-rDNA regions) by metaphase, in contrast to condensins.
We addressed the timings that Top2 and condensins are required for proper chromosome segregation during the cell cycle. *smc2-8* (T3829), *ycg1-2* (T3992), *top2-4* (T3794) and wild-type control cells (T3790; see their genotypes in Fig 3 and 5A) were treated as follows: 'G1' temperature-shift; cells were treated as in Fig 3. 'M' metaphase temperature-shift; cells were treated as above but shifted to 35 ˚C during metaphase arrest (30 min prior to Cdc20 re-expression). The time required for completion of *HIS*-locus segregation (time 0 as defined in Fig 3) towards the bud was then scored in both conditions. *n.s.*= not significant.
Results. This data suggests that condensins cannot complete their roles in chromosome segregation by metaphase, in contrast to Top2.

Note (for Fig 5 and Fig S5): The ectopic expression of *Chlorella* virus topoisomerase II does not rescue a segregation defect of a non-rDNA region in *ycg1-2*.

It was recently reported that, in budding yeast, the defect in rDNA segregation in condensin mutants is rescued by the ectopic expression of *Chlorella* virus topoisomerase II (CV-topII; (D'Ambrosio et al., 2008)). This result was interpreted that the evolutionarily distant topoisomerase II may have evaded condensin-dependent regulation for rDNA resolution. To address whether the ectopic expression of CV-topII also rescues the segregation defect of chromosome XV in *ycg1-2*, we expressed it from a galactose-inducible promoter (*GAL1-10* promoter) and observed the behaviour of *CEN*, *HIS* and *TEL* GFP dots on this chromosome. After its expression we still found a similar defect in the segregation of chromosome XV during anaphase. Thus the ectopic expression of CV-topII did not rescue a segregation defect of a non-rDNA region in *ycg1-2*. This is consistent with our conclusion that condensins' role in chromosome segregation of non-rDNA regions is not limited to resolution of sister chromatids.

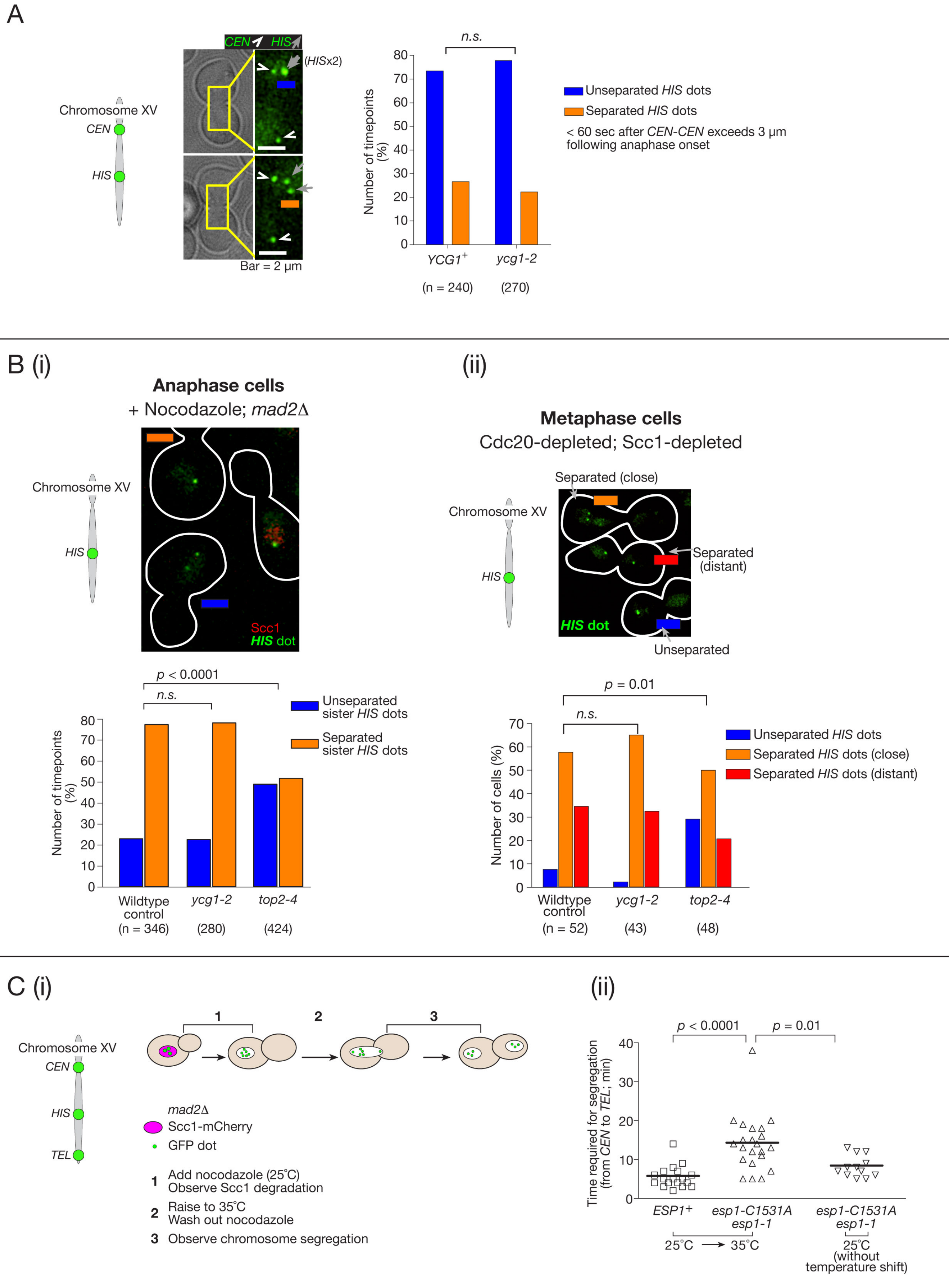

Supplemental Fig S7

Figure S7 (associated with Figure 7)

A. The amount of residual cohesion between sister chromatids is similar between *YCG1*⁺ and *ycg1-2* cells, soon after anaphase onset and before chromosome stretching/recoiling reaches the region.

We aimed to compare the amount of residual sister chromatid cohesion at a chromosome locus (we chose the *HIS* locus for this purpose) between *YCG1*$^+$ and *ycg1-2* cells. We sought to make this comparison over a certain period after the onset of anaphase but before the regional chromosome stretching/recoiling involved the *HIS* locus. We carried out a similar experiment to Fig 3A, but using a strain with *CEN* and *HIS* dots but without the *TEL* dot. The minimum time required for *HIS* segregation to begin (after the *CEN-CEN* distance exceeded 3 μm) was determined to be 60 sec (see supplemental experimental procedures). Using the same data set, we then scored the number of cells and the time points, in which the sister *HIS* dots were associated over the 60 sec after anaphase onset.

Results. 4 *YCG1*$^+$ cells (out of 16) and 6 *ycg1-2* cells (out of 18) showed continuous association of the *HIS* dots over the period. *YCG1*$^+$ and *ycg1-2* cells showed *HIS* dot separation in 27 % (64/240) and 22 % (60/270) of time points (shown in the graph), respectively, over the 60 sec. These data did not show significant difference between *YCG1*$^+$ and *ycg1-2* cells, suggesting that the amount of residual sister chromatid cohesion does not change in the *ycg1-2* mutant during the initial stage of anaphase (before chromosome stretching/recoiling involves a relevant chromosome arm region).

B. *top2-4* mutant cells, but not *ycg1-2* mutants, show defects in resolution between sister chromatids during anaphase (i) and metaphase (ii).

i) *ycg1-2* (T7173), *top2-4* (T7174) and *TOP2*$^+$ *YCG1*$^+$ control (T7172) cells with *mad2*Δ *HIS-tetOs TetR-GFP SCC1-4xmCherry* were treated with α factor at 25 ˚C, then released into fresh medium containing nocodazole at 35 ˚C. After 70 min, GFP and mCherry images were acquired every 4 min for 92 min at 35 ˚C. Representative cells (top) and separation of sister GFP dots, scored at each time point after cells entered anaphase i.e. after Scc1 signals were reduced in the nuclei (bottom). n = number of observed time points (in 21-26 cells of each yeast strain).

Results. When *top2-4 mad2*Δ cells entered anaphase after nocodazole treatment they showed a reduced separation rate of the *HIS* chromosome arm locus, compared with *TOP2*$^+$ *YCG1*$^+$ *mad2*Δ cells, presumably due to remaining catenation between sister chromatids. By contrast, *ycg1-2 mad2*Δ cells did not show such reduction.

ii) *top2-4* (T7063), *ycg1-2* (T7061), and wild-type control (*TOP2*$^+$ *YCG1*$^+$; T6984) cells with *PGAL-CDC20 PGAL-SCC1 TetR-GFP HIS-tetOs* were arrested with α factor treatment at 25 ˚C (for 3 hours), then released into fresh medium at 35 ˚C and arrested at metaphase by Cdc20 depletion. In order to remove sister chromatid cohesion, transcription of *PGAL-SCC1* was inhibited by incubating cells in glucose-containing medium during α factor treatment (the last 2 hours 15 min) and after the release from it. After 3 hours from the release from α factor treatment, GFP images were acquired every 20 sec for 5 min. In the absence of sister chromatid cohesion, separation of sister *HIS*-GFP dots during metaphase arrest should be dependent on successful resolution between sister chromatids around the *HIS* locus. We categorized separation of sister *HIS*-GFP dots in individual cells as defined in supplemental experimental procedures. *n* = number of analyzed cells. *n.s.*= not significant.

Results. When we compared the separation rate of the *HIS* locus in cohesin-depleted and metaphase-arrested (Cdc20-depleted) cells, *top2-4* cells showed a reduced separation rate relative to 'wild-type' *TOP2*$^+$ *YCG1*$^+$ cells. However *ycg1-2* cells did not show such reduction. Thus, the defect in sister resolution was not detected in the *ycg1-2* mutant, which showed the severest defect in chromosome segregation (at least for chromosome XV) among condensin-*ts* mutants that we tried (*ycg1-2*, *smc2-8*, *ycg1-10* and *brn1-9*; data not shown).

C. Separase cleavage activity is required during anaphase for efficient chromosome segregation.

We addressed whether the activity of separase (Esp1 in budding yeast) continues to be required

after anaphase onset to complete sister chromatid separation and segregation. We used *esp1-C1531A*, which is an *esp1* catalytically inactive mutant that can support anaphase spindle integrity but not Scc1 cleavage (Sullivan and Uhlmann, 2003). Because the C1531A mutant is not viable, the *esp1-1* mutant (Baum et al., 1988; Ciosk et al., 1998) was combined to keep cells alive at 25 ˚C. We inactivated *esp1-1* by shifting the temperature to 35 ˚C after entry into anaphase (after Scc1 was reduced in nuclei) but before chromosome segregation (i) by the following procedure. *esp1-C1531A esp1-1* (T7406) and *ESP1*$^+$ (T7162) cells with *mad2Δ SCC1-4xmCherry TetR-GFP tetOs* (integrated at three loci as in Fig 1A) were treated with α factor at 25 ˚C, then released into fresh medium containing nocodazole and incubated for 80 min, followed by acquisition of GFP and mCherry images every 3 min for 45 min. Cells were then released into fresh medium at 35 ˚C without nocodazole, followed by image acquisition every min for 1 hour. As a control, T7406 cells were also treated similarly but without the temperature shift. In individual cells that showed both Scc1 reduction before the temperature shift and subsequent bi-orientation of sister *CEN*s (GFP-labelled) after nocodazole washout, time was measured from *CEN* segregation to *TEL* segregation (to each cell body; *CEN-CEN* and *TEL-TEL* distances became > 3 μm, respectively). Thick lines indicate mean values. The *mad2*-deleted cells, treated with nocodazole, entered anaphase with their mitotic spindle disrupted (as in Fig S4D). Subsequent nocodazole washout allowed cells to form a bipolar spindle, followed by segregation of sister *CEN*s to the opposite spindle poles in 21/146 *ESP1*$^+$ cells and 22/103 *esp1*-C1531A cells (difference not significant), while in the other cells both sisters were pulled towards one pole. In cells that showed bi-orientation of sister *CEN*s, we compared the behaviour of chromosome XV in *ESP1*$^+$ and *esp1*-C1531A mutant cells.
Results. Following segregation of sister *CEN*s, the *esp1* catalytically inactive mutant showed segregation of *HIS* and *TEL* loci to each daughter nucleus, accompanied by regional chromosome stretching and recoiling (similarly to Fig 1A). However, it took significantly longer for the mutant to complete *TEL* segregation in comparison to *ESP1*$^+$ wild-type (ii). Thus, at least in this experimental condition, the catalytic activity of Esp1 is still required after anaphase onset for efficient chromosome segregation.

Supplemental Note for Power Spectrum Analyses
(associated with Figure 6C)

Discrete Fourier transforms of chromosome length time series, $x_1,...,x_N$, were calculated using the Cooley-Tukey algorithm included in Matlab. All signals were re-centered so that the dc (direct current) component was set to zero.

$$X_k = \frac{1}{N}\sum_{n=1}^{N} x_n \exp\left(-2\pi i(k-1)\frac{n-1}{N}\right) \qquad (1)$$

The power spectrum was then calculated by taking the square of the magnitude of the Fourier transform

$$P_k = 2X_k X_k^* \qquad (2)$$

so that the integral of the power spectrum $\sum_{k}^{N/2} P_k$ is equal to the variance of the signal (the summation is only carried to *k*=*N*/2 because of the Nyquist criterion for discrete signals). The power spectra of the eight experimental replicates were calculated independently and combined to give the mean power spectra for both the *YCG1*$^+$ wild-type and *ycg1-2* mutants. The error estimates were obtained by carrying out bootstrap re-sampling of the eight independent replicates and sorting the resulting spectra according to the total power output. The 90% confidence intervals therefore correspond to the samples in the 5th and 95th centile of the bootstrap replicates (Efron and Tibshirani, 1994).

Several functions were fitted to the power spectra using the unconstrained non-linear optimization function *fminsearch* included with Matlab, with minimization of the log-mean squared error. The first of these (model 1) is passive diffusion, which applies to objects that are not actively transported (or constrained) by internal structures within the cell. This model applies to freely-diffusing particles, such as the protein components of intra-cellular signaling cascades (Slaughter et al., 2007).

$$P(f) = \frac{D}{\pi^2} f^{-2} \qquad (3)$$

where *D* is the diffusion coefficient.

It has been found, in both *in vivo* and *in vitro* experiments, that metazoan anaphase chromosomes behave as Hookean springs with an extension that is proportional to the applied force (Marko, 2008; Nicklas, 1988). At smaller length scales, where fluctuations due to Brownian motion are appreciable, fluctuations in the length of the chromosome can be used to infer the spring stiffness and the magnitude of the damping forces indirectly (Meyhofer and Howard, 1995) by fitting a Lorentzian function to the power spectrum. This function is of the form

$$P(f) = \frac{4kT\gamma}{\kappa^2}\frac{1}{1+(2\pi f\gamma/\kappa)^2} \qquad (4)$$

where g is the drag co-efficient, k, the elastic constant, *k* is the Boltzmann constant and *T* the temperature. This situation corresponds to an elastic model of the anaphase chromosome as depicted below.

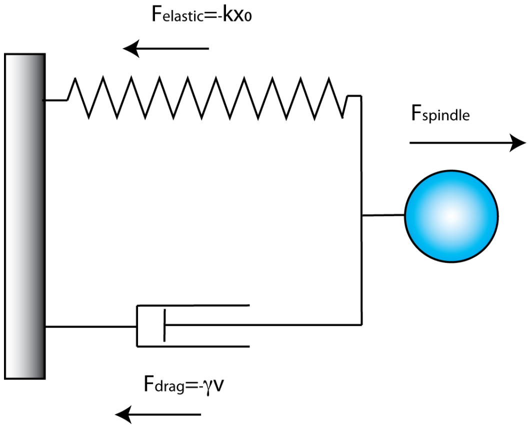

(Particle confined in a harmonic potential. At low Reynolds numbers the mass of the particle can be neglected.)

Finally model 3 is a general power-law function, which has an identical form to model 1, except that the exponent l is a fitting parameter

$$P(f) = Af^{\lambda} \tag{5}$$

where *A* is another fitting parameter without a direct physical interpretation. The estimates for these two parameters for *YCG1*$^{+}$ cells are l=1.30±0.01 and $A = 3.14 \pm 1.10 \times 10^{-5}$. It is notable that the predictions of model 1 (l=2) are not within the error bounds of model 3.

The co-efficient of determination R^2 in log-space is 0.77 for model 1, 0.72 for model 2 and 0.85 for model 3 in *YCG1*$^{+}$ cells. The log-mean-squared error is also lower for the power law fit than the other two models.

Further evidence for rejection of the passive spring (model 2) is provided by the estimates for the constant *k* which measures the spring stiffness. The estimate of the stiffness is decreased by a factor of 3 in the *YCG1*$^{+}$ cells compared with the mutant condensin, which cannot easily be reconciled with the activity of condensin homologues observed in bacteria (Cui et al., 2008) and of condensin II in higher eukaryotes (Strick et al., 2004), unless the yeast condensin is involved in actively compacting the chromosome.

Supplemental Experimental Procedures

Yeast Genetics and Molecular Biology

The background of yeast strains (W303) and methods for yeast culture, treatment with α-factor and nocodazole, their subsequent wash-out, and FACS DNA content analysis were as described previously (Amberg et al., 2005; Tanaka et al., 2007).

Constructs of *TetR-GFP, SCC1-18×myc* (Michaelis et al., 1997), *TetR-3×CFP* (Bressan et al., 2004), *GFP-lacI* (Straight et al., 1996), *PDS1-18×myc* (Ciosk et al., 1998), *PGAL-CDC6* (Piatti et al., 1996), *PMET-CDC20* (Uhlmann et al., 2000), *PGAL-CDC20* (Lim et al., 1998), *PGAL-SCC1* (Uhlmann and Nasmyth, 1998), *PGAL-R* (Raghuraman et al., 1997) and *PGAL-CEN3* (Hill and Bloom, 1987) were previously described. Mutant alleles *ycg1-2* (Lavoie et al., 2002), *smc2-8* (Freeman et al., 2000), *top2-4* (Holm et al., 1985), *esp1-1* (Baum et al., 1988) and *esp1*-C1531A (Sullivan and Uhlmann, 2003) were previously reported.

To mark *CEN15* (2 kb left of *CEN15*), *TEL15R* (18 kb from *TEL15R*) and *MCH5* (between *MCH5* and *SLY41*), 112×*tetO*s of 5.6 kb (Michaelis et al., 1997) was inserted into a plasmid containing both a 500-700 bp genomic fragment (spanning each insertion site) and an auxotroph marker (or an antibiotic marker), which was subsequently cut within the genomic fragment and inserted into each genome locus. To mark *HIS3* and *ADE2* loci, 112×*tetO*s and 256×*lacO*s of 10.1 kb (Straight et al., 1996) were inserted into plasmids containing *HIS3* and *ADE2*, respectively, which were subsequently cut within the auxotroph marker and inserted into each locus.

A plasmid containing *3×CFP-LacI* (pT1034) was constructed by replacing GFP with three tandem copies of CFP on the *GFP-lacI* plasmid (Straight et al., 1996). *SMC4* and *YCG1* were tagged with three tandem copies of *GFP*; *NET1* and *SCC1* with four tandem copies of *mCherry* (Shaner et al., 2004); and *NDC80* with three tandem copies of *CFP* at their C-termini at their original gene loci by a one-step PCR method as described previously (Maekawa et al., 2003; Tanaka et al., 2007), using cassettes for PCR, containing 3×GFP, 4×mCherry and 3×CFP; pSM1023 (Maekawa et al., 2003), pT909 and pT769, respectively. pT909 was constructed by multiplying the *mCherry* gene in pKS391 (Snaith et al., 2005). Strains with the tagged genes grew normally at temperatures used in this study.

SCC1 and *SMC2* were tagged with an auxin-inducible degron (*aid*) at their C-termini at their original gene loci and their protein degradation was facilitated within cells carrying rice *Oryza sativa TIR1* (*osTIR1*, under the control of a constitutive *ADH1* promoter) in the presence of auxin NAA (1-naphthaleneacetic acid), as described previously (Nishimura et al., 2009). NAA was dissolved in DMSO (4 mM) and then added to culture media to the final concentration of 20 μM, while the same amount of DMSO was added for a control without NAA. To quantify Scc1-aid and Smc2-aid proteins in Figs S2D and S4D, an antibody against the *aid* tag (Nishimura et al., 2009) was used in Western blots (in which Cdc28 was also detected using an anti-Cdc28 antibody [Santa Cruz] as a loading control).

To flip out *CEN12* by induced homologous recombination, *CEN12* was replaced with *CEN3* flanked by two *RS*s (recombination sites for *Z. rouxii* R recombinase; Matsuzaki et al., 1990) on chromosome XII as shown in Fig S4A (i). To make a dicentric chromosome, *PGAL-CEN3* (Hill and Bloom, 1987) was inserted at the

position on chromosome IV, as indicated in Fig 6A (i), using T3959 strain (containing *tetO*s and *lacO*s on this chromosome; Kitamura et al., 2006).

Cells were cultured at 25 ºC in YP medium (Amberg et al., 2005) containing 2 % glucose, unless otherwise stated. To activate or suppress the *GAL1-10* promoter (*PGAL*), cells were incubated in medium containing galactose (plus raffinose) or glucose (or raffinose when it is activated again), respectively (2 % each). To activate or suppress the *MET3* promoter (*PMET*), cells were incubated in methionine-drop-out medium or YP medium plus additional 2 mM methionine (both with appropriate carbon source), respectively.

Microscopy

The procedures for time-lapse fluorescence microscopy were described previously (Tanaka et al., 2007). Time-lapse images were collected at 25 ºC unless otherwise stated. For image acquisition, we used a DeltaVision RT microscope (Applied Precision), an UPlanSApo 100× objective lens (Olympus; NA 1.40), SoftWoRx software (Applied Precision), and either a CoolSnap HQ (Photometrics) or Cascade II 512B (Roper Scientific) CCD camera. We acquired 7-9 (0.7 mm apart) z-sections, which were subsequently deconvoluted, projected to two-dimensional images and analyzed with SoftWoRx and Volocity (Improvision) software. CFP, GFP and mCherry signals were discriminated using the 89006 multi-band filter set (Chroma). To collect GFP signals alone, the FITC filter (Chroma) was used.

Analyzing Dynamics of Chromosome Behaviours

To evaluate the distance and speed concerning chromosome dynamics in live-cell imaging, the distance along the z-axis, as well as the distance on a projected image (x-y), was taken into account. To measure the speed of a GFP dot's movement, the velocity in shortening of a relevant distance (e.g. *CEN-HIS*, *CEN-TEL*) was measured after being fitted to linear regression ($R^2 > 0.9$) over a change larger than 1.5 µm. When we scored % of cells or time points with separated sister GFP dots, in a small number of cells (< 1 %) we found two dots, which should be attributed to chromosome mis-segregation in previous cell cycles rather than sister separation in the present cycle, based on their GFP intensity (Kitamura et al., 2006); such cells were excluded from scoring.

Statistical analyses were carried out using Prism (Graph Pad) software, by choosing the unpaired *t*-test (Figs 1B-iii, 2B-iv, 3C, 4B-iv&v, 5B-ii, 6B-iii, 6C-ii, 7A-iii, S1B-ii&iii, S7C-ii), Fisher's exact test for contingency table (Figs 2B-iii, 7A-ii, S2A-ii&iii, S4E-iii, S7A, S7B-i) and the *chi*-square test for trend (Figs 3B, 4B-iii, 7B, S5D, S7B-ii). See the methods for power spectra analyses in Supplemental Note for Power Spectrum Analyses.

To address whether the presence of *tetOs*/tetR-GFP on a chromosome delays the chromosome completing segregation in Figs 1A and B, we compared segregation timing of *TEL* GFP-dot on chromosome XV (after the distance between sister *CEN*s exceeded 3 µm) in the presence and absence of *tetOs*/tetR-GFP on other chromosome loci; i.e. in strains with 1) only *CEN* and *TEL* dots, 2) *CEN, HIS* and *TEL* dots (see Fig 1A i), and 3) *CEN, HIS, MCH* and *TEL* dots (see Fig 1B i). If the presence of *tetOs*/tetR-GFP on a chromosome delays the chromosome segregation, we would expect a further delay in *TEL* segregation in the strains 2) and 3) relative to the strain 1). However there was no such delay observed (data not shown). It is therefore unlikely that the presence of *tetOs*/tetR-GFP on a chromosome arm causes a delay in segregation of the chromosome arm.

In Fig 1B, one can argue that larger stretching per 10 kb for *HIS-MCH* and *MCH-TEL* than *HIS-TEL*, may not be due to stretching being limited to a short chromosomal region, but rather due to a particular position of *HI*S, *MCH* and *TEL* loci in anaphase. Indeed, in theory, *HIS-MCH* and *MCH-TEL* may show stretching simultaneously (i.e. *HIS-TEL* region is stretched uniformly at a given time) but these three loci may locate at the vertex of a triangle. This would indeed explain the result in Fig 1B. However this possibility was unlikely because all three loci (*HIS*, *MCH* and *TEL*) located almost along the axis between two sister CENs when their interval regions showed stretching in anaphase. To exclude this possibility rigorously, *HIS*, *MCH* and *TEL* loci were visualized in the same cell. The *HIS-MCH* extension occurred earlier than the *MCH-TEL* extension (data not shown), assuming that segregation took place from *CEN*-proximal to –distal along a chromosome arm. Thus the above possibility was excluded.

In Fig 2B, the distances between sister *CEN*s and sister *ADE*s did not exceed 3 and 1.5 μm, respectively, before Cdc20 was re-expressed or after metaphase was extended for additional 45 min (during which, NAA was added to the culture medium) as a control. In other words, Scc1 depletion alone was not sufficient to cause such events; anaphase onset was required. From the results in Fig 2B, we conclude that the residual cohesion is at least partially dependent on cohesins, which rules out that residual cohesion in early anaphase is simply an artefact due to stickiness between GFP-labelled sister loci (Fuchs et al., 2002).

In Fig 6A, we made and identified an unreplicated chromosome with two centromeres, as follows. An unreplicated chromosome with two centromeres was generated in the cells where the only *CDC6* was under the control of the *GAL* promoter (Piatti et al., 1996), the only *CDC20* was under the control of the *MET* promoter (Uhlmann et al., 2000), and the ectopic *CEN3* (e*CEN3*: inserted on the left arm of chromosome IV) was under the control of the *GAL* promoter (Hill and Bloom, 1987). GFP and CFP dots were inserted between e*CEN3* and the authentic *CEN4* (Fig 6A i). Cells were first arrested in metaphase by shutting off *CDC20* expression, then treated briefly (30 min) with glucose (without galactose) to turn off the *GAL* promoter (causing inhibition of *CDC6* expression as well as the activation of e*CEN3*) during metaphase arrest, and subsequently released to anaphase by re-expressing *CDC20* with the *GAL* promoter still turned off. When e*CEN3* and *CEN4* on the same chromatid attached to microtubules from the opposite spindle poles (bi-orientation) in this circumstance, the chromosome region between GFP and CFP showed stretching and shortening repeatedly, which led to chromosome breakage after a delay in cytokinesis (data not shown). Chromosome breakage resulted in two GFP and/or CFP dots in the same cell (Brock and Bloom, 1994) (even if the two dots overlapped we could identify them because GFP/CFP intensity was higher than a single dot) and/or failure in bi-orientation of e*CEN3-CEN4* in the next cell cycle, depending on the position of breakage; such cells were recognized by time-lapse microscopy and excluded from further study. We also confirmed by FACS analyses that, after release from the metaphase arrest, cells with 1C DNA content accumulated (Fig 6A ii), thus DNA replication was indeed inhibited. We then focused on cells where e*CEN3* and *CEN4* showed bi-orientation on the spindle in the next cell cycle (i.e. the chromosome region between GFP and CFP dots showed stretching and shortening, repeatedly) and analyzed their motion after cells presumably entered anaphase (after cells showed the elongated nucleus between the bud and mother cell body; the nucleus was visualized by a small amount of GFP-LacI, not bound to *lac* operators) but before the chromosome was broken.

In Fig 7A, chromosomes were fixed and immobilized immediately after cell lysis, as described previously (Tanaka et al., 1997). Subsequently they were immuno-stained

using an anti-myc antibody (9E10) and Alexa Fluor 594 anti-mouse IgG (Molecular Probe). We identified *CEN* and *TEL* dots on fixed chromosome samples, as follows. DNA was stained with DAPI and we found that the shape of the DAPI staining changes very similarly to the shape of the nucleus in corresponding live cells; the shape was round in metaphase (Cdc20-depleted cells), slightly elongated in early anaphase, further elongated in mid-anaphase and separated to two masses in late anaphase (progressively after Cdc20 re-expression). We therefore presumed that the positions of *CEN* and *TEL* dots on fixed chromosomes might reflect those in corresponding live cells. For example, we presumed that *TEL* dots do not separate while *CEN* dots show separation on fixed chromosomes in metaphase; indeed one dot showed high intensity while two dots showed low intensity. It was also presumed that, on fixed chromosomes, 1) during early anaphase (the distance between sister *CEN*s < 4 μm) sister *TEL* dots are either not separated or are in close proximity to each other (< 1 μm), and that 2) during mid-late anaphase (the distance between sister *CEN*s > 4 μm) sister *CEN*s locate at the opposite end of the elongated nucleus, as observed in live cells (see Fig 1A and S1). Based on these presumptions, we could identify *CEN* and *TEL* dots (both labeled with GFP) on fixed chromosomes, without ambiguity in >95 % cases. Such identification was rationalized by several findings; for example, 1) the distance between sister '*CEN* dots' was enlarged only after anaphase was induced (Fig 7A ii, bottom) and its distribution in a cell population after anaphase onset was similar to that of live cells (data not shown), 2) segregation of '*TEL* dots' to the opposite sides of the nucleus was observed more frequently after the '*CEN*-*CEN* distance' was enlarged in *YCG1*$^+$ cells (Fig 7A ii), and 3) segregation of '*TEL* dots' were found less frequently during anaphase in *ycg1-2* cells than in *YCG1*$^+$ cells (Fig 7A ii). Subsequently, based on the distance between sister *CEN*s, we classified the fixed chromosome samples as early or mid-late anaphase (Fig 7A ii, bottom). This was rationalized by the findings that 1) the distance between sister *CEN* dots was enlarged only after anaphase was induced (Fig 7A ii, bottom), similarly to live cells and with similar kinetics between *YCG1*$^+$ and *ycg1-2* cells and 2) segregation of *TEL* dots to the opposite sides of the nucleus was observed more frequently in the defined mid-late anaphase of *YCG1*$^+$ cells than in early anaphase (Fig 7A ii).

In Fig S7A, we aimed to compare the amount of residual sister chromatid cohesion at a chromosome locus (we chose the *HIS* locus for this purpose) between *YCG1*$^+$ and *ycg1-2* cells. We sought to make this comparison over a certain period after the onset of anaphase but before the regional chromosome stretching/recoiling involved the *HIS* locus. It was inappropriate to set this period differently in individual cells, as this period is generally longer in *ycg1-2* cells than in *YCG1*$^+$ cells; for example, if residual cohesion reduces over time it would hamper fair comparison. Therefore we first sought to determine the minimum time required for the sister *HIS* dots to start segregation (the distance between sister *HIS* loci first exceeded 2 μm; note that this distance never exceeded 2 μm even if they separated prior to the start of segregation) once the *CEN*-*CEN* distance first exceeded 3 μm (i.e. anaphase onset) in *YCG1*$^+$ cells. For this, we carried out a similar experiment to Fig 3A, but using a strain with *CEN* and *HIS* dots but without the *TEL* dot. The minimum time required for *HIS* segregation to begin was determined to be 60 sec. Using the same data set, we then scored the number of cells and the time points, in which the sister *HIS* dots were associated over the 60 sec after anaphase onset.

In Fig S7B ii, we categorized separation of sister *HIS*-GFP dots in individual cells as follows: 1) Unseparated; sister GFP dots separated at < 60 % of time points of observation, 2) Separated (close); sister GFP dots separated at > 60 % of time points of observation but their maximum distance apart was < 2.75 µm, 3) Separated

(distant); sister GFP dots separated at > 60 % of time points of observation and their maximum distance apart was > 2.75 μm.

Notes about Spindle Assembly Checkpoint in Condensin Mutants

It is reported that a fraction of condensin mutant cells show a delay in metaphase with dependence on spindle-assembly checkpoint (SAC; (Yong-Gonzalez et al., 2007). To address if SAC satisfaction is delayed in condensin mutants in our experimental conditions (Figs 3A and 4B), we compared the timing of anaphase onset by evaluating the start of Scc1 reduction in the nucleus or the elongation of sister *CEN* distance in wild-type, *smc2-8*, *ycg1-2* and *smc2-aid* cells. We also compared the timing of anaphase onset in *ycg1-2* cells in the presence and absence of *MAD2*. In both comparisons, anaphase onset was very similar between the cells (data not shown). Therefore we conclude that, in these condensin mutants, SAC becomes satisfied prior to anaphase onset without a delay, similarly to wild-type cells, at least in our experimental conditions.

On the other hand, it was still formally possible that condensin defects caused sustained residual sister chromatid cohesion in early anaphase due to failure in keeping SAC satisfied (SAC may be still active during early anaphase; Khodjakov and Rieder, 2009). For example, chromosome recoiling by condensins may generate weak tension at kinetochores and this may be necessary to keep SAC satisfied during early anaphase. However, even if this were the case, this was not the reason for sustained residual cohesion or segregation defects, found in condensin mutants, because *ycg1-2* cells showed defects in chromosome recoiling and sister chromatid separation, similarly in the presence and absence of *MAD2* (data not shown).

Supplemental References

Amberg, D.C., Burke, D.J., and Strathern, J.N. (2005). Methods in yeast genetics (CSHL press).

Baum, P., Yip, C., Goetsch, L., and Byers, B. (1988). A yeast gene essential for regulation of spindle pole duplication. Mol Cell Biol *8*, 5386-5397.

Bressan, D.A., Vazquez, J., and Haber, J.E. (2004). Mating type-dependent constraints on the mobility of the left arm of yeast chromosome III. J Cell Biol *164*, 361-371.

Brock, J.A., and Bloom, K. (1994). A chromosome breakage assay to monitor mitotic forces in budding yeast. J Cell Sci *107 (Pt 4)*, 891-902.

Ciosk, R., Zachariae, W., Michaelis, C., Shevchenko, A., Mann, M., and Nasmyth, K. (1998). An ESP1/PDS1 complex regulates loss of sister chromatid cohesion at the metaphase to anaphase transition in yeast. Cell *93*, 1067-1076.

Cui, Y., Petrushenko, Z.M., and Rybenkov, V.V. (2008). MukB acts as a macromolecular clamp in DNA condensation. Nat Struct Mol Biol *15*, 411-418.

D'Ambrosio, C., Kelly, G., Shirahige, K., and Uhlmann, F. (2008). Condensin-dependent rDNA decatenation introduces a temporal pattern to chromosome segregation. Curr Biol *18*, 1084-1089.

Efron, B., and Tibshirani, R. (1994). An Introduction to the Bootstrap (CRC Press).

Freeman, L., Aragon-Alcaide, L., and Strunnikov, A. (2000). The condensin complex governs chromosome condensation and mitotic transmission of rDNA. J Cell Biol *149*, 811-824.

Fuchs, J., Lorenz, A., and Loidl, J. (2002). Chromosome associations in budding yeast caused by integrated tandemly repeated transgenes. J Cell Sci *115*, 1213-1220.

Hill, A., and Bloom, K. (1987). Genetic manipulation of centromere function. Mol Cell Biol *7*, 2397-2405.

Holm, C., Goto, T., Wang, J.C., and Botstein, D. (1985). DNA topoisomerase II is required at the time of mitosis in yeast. Cell *41*, 553-563.

Khodjakov, A., and Rieder, C.L. (2009). The nature of cell-cycle checkpoints: facts and fallacies. J Biol *8*, 88.

Kitamura, E., Blow, J.J., and Tanaka, T.U. (2006). Live-cell imaging reveals replication of individual replicons in eukaryotic replication factories. Cell *125*, 1297-1308.

Lavoie, B.D., Hogan, E., and Koshland, D. (2002). In vivo dissection of the chromosome condensation machinery: reversibility of condensation distinguishes contributions of condensin and cohesin. J Cell Biol *156*, 805-815.

Lim, H.H., Goh, P.Y., and Surana, U. (1998). Cdc20 is essential for the cyclosome-mediated proteolysis of both Pds1 and Clb2 during M phase in budding yeast. Curr Biol *8*, 231-234.

Maekawa, H., Usui, T., Knop, M., and Schiebel, E. (2003). Yeast Cdk1 translocates to the plus end of cytoplasmic microtubules to regulate bud cortex interactions. Embo J *22*, 438-449.

Marko, J.F. (2008). Micromechanical studies of mitotic chromosomes. Chromosome Res *16*, 469-497.

Matsuzaki, H., Nakajima, R., Nishiyama, J., Araki, H., and Oshima, Y. (1990). Chromosome engineering in Saccharomyces cerevisiae by using a site-specific recombination system of a yeast plasmid. J Bacteriol *172*, 610-618.

Meyhofer, E., and Howard, J. (1995). The force generated by a single kinesin molecule against an elastic load. Proc Natl Acad Sci U S A *92*, 574-578.

Michaelis, C., Ciosk, R., and Nasmyth, K. (1997). Cohesins: chromosomal proteins that prevent premature separation of sister chromatids. Cell *91*, 35-45.

Musacchio, A., and Salmon, E.D. (2007). The spindle-assembly checkpoint in space and time. Nat Rev Mol Cell Biol *8*, 379-393.

Nicklas, R.B. (1988). The forces that move chromosomes in mitosis. Annu Rev Biophys Biophys Chem *17*, 431-449.
Nishimura, K., Fukagawa, T., Takisawa, H., Kakimoto, T., and Kanemaki, M. (2009). An auxin-based degron system for the rapid depletion of proteins in nonplant cells. Nat Methods *6*, 917-922.
Piatti, S., Bohm, T., Cocker, J.H., Diffley, J.F., and Nasmyth, K. (1996). Activation of S-phase-promoting CDKs in late G1 defines a "point of no return" after which Cdc6 synthesis cannot promote DNA replication in yeast. Genes Dev *10*, 1516-1531.
Raghuraman, M.K., Brewer, B.J., and Fangman, W.L. (1997). Cell cycle-dependent establishment of a late replication program. Science *276*, 806-809.
Shaner, N.C., Campbell, R.E., Steinbach, P.A., Giepmans, B.N., Palmer, A.E., and Tsien, R.Y. (2004). Improved monomeric red, orange and yellow fluorescent proteins derived from Discosoma sp. red fluorescent protein. Nat Biotechnol *22*, 1567-1572.
Shimada, K., and Gasser, S.M. (2007). The origin recognition complex functions in sister-chromatid cohesion in Saccharomyces cerevisiae. Cell *128*, 85-99.
Slaughter, B.D., Schwartz, J.W., and Li, R. (2007). Mapping dynamic protein interactions in MAP kinase signaling using live-cell fluorescence fluctuation spectroscopy and imaging. Proc Natl Acad Sci U S A *104*, 20320-20325.
Snaith, H.A., Samejima, I., and Sawin, K.E. (2005). Multistep and multimode cortical anchoring of tea1p at cell tips in fission yeast. EMBO J *24*, 3690-3699.
Straight, A.F., Belmont, A.S., Robinett, C.C., and Murray, A.W. (1996). GFP tagging of budding yeast chromosomes reveals that protein-protein interactions can mediate sister chromatid cohesion. Curr Biol *6*, 1599-1608.
Strick, T.R., Kawaguchi, T., and Hirano, T. (2004). Real-time detection of single-molecule DNA compaction by condensin I. Curr Biol *14*, 874-880.
Sullivan, M., and Uhlmann, F. (2003). A non-proteolytic function of separase links the onset of anaphase to mitotic exit. Nat Cell Biol *5*, 249-254.
Tanaka, K., Kitamura, E., Kitamura, Y., and Tanaka, T.U. (2007). Molecular mechanisms of microtubule-dependent kinetochore transport toward spindle poles. J Cell Biol *178*, 269-281.
Tanaka, T., Knapp, D., and Nasmyth, K. (1997). Loading of an Mcm protein onto DNA replication origins is regulated by Cdc6p and CDKs. Cell *90*, 649-660.
Uhlmann, F., and Nasmyth, K. (1998). Cohesion between sister chromatids must be established during DNA replication. Curr Biol *8*, 1095-1101.
Uhlmann, F., Wernic, D., Poupart, M.A., Koonin, E.V., and Nasmyth, K. (2000). Cleavage of cohesin by the CD clan protease separin triggers anaphase in yeast. Cell *103*, 375-386.
Yong-Gonzalez, V., Wang, B.D., Butylin, P., Ouspenski, I., and Strunnikov, A. (2007). Condensin function at centromere chromatin facilitates proper kinetochore tension and ensures correct mitotic segregation of sister chromatids. Genes Cells *12*, 1075-1090.
