# Supplementary figures and images for: Condensins Promote Chromosome Recoiling during Early Anaphase to Complete Sister Chromatid Separation
Source: Dev Cell. 2010 Aug 17;19(2):232–44. doi: 10.1016/j.devcel.2010.07.013 (PMC2938479; doi:10.1016/j.devcel.2010.07.013)

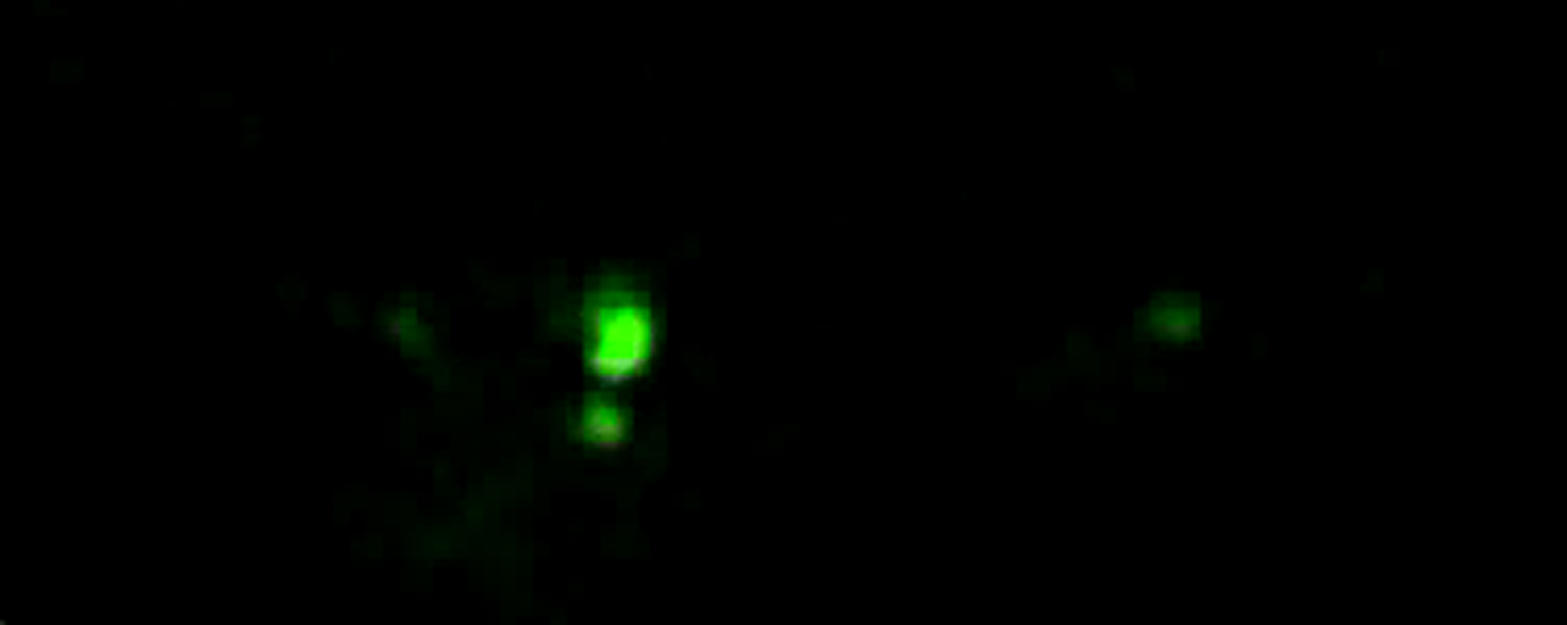

Supplement: Movie S1. Movie of the Cell Shown in Figure 1A — The speed of all movies is 40 times faster than the actual movements. [file mmc2.jpg]

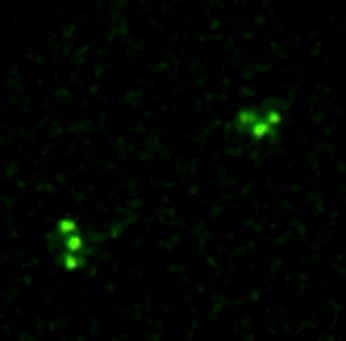

Supplement: Movie S2. Movie of the Cell Shown in Figure 3A (wild-type) — The speed of all movies is 40 times faster than the actual movements. [file mmc3.jpg]

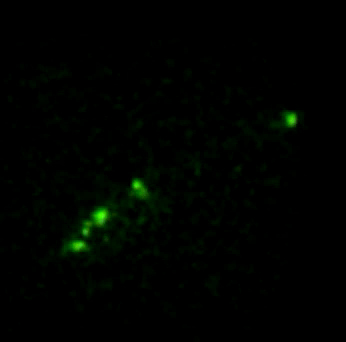

Supplement: Movie S3. Movie of the Cell Shown in Figure 3A (smc2-8) — The speed of all movies is 40 times faster than the actual movements. [file mmc4.jpg]

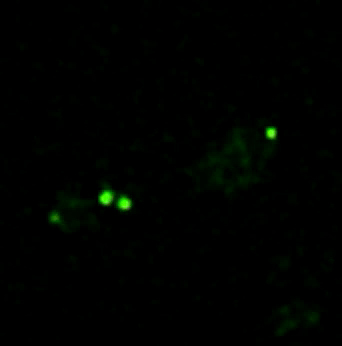

Supplement: Movie S4. Movie of the Cell Shown in Figure 3A (ycg1-2) — The speed of all movies is 40 times faster than the actual movements. [file mmc5.jpg]

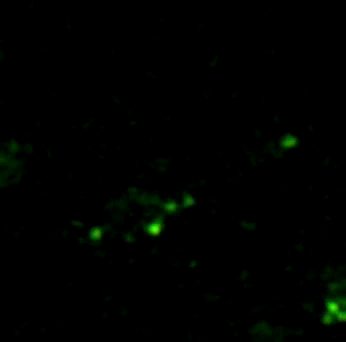

Supplement: Movie S7. Movie of Cell #3 (top2-4) Shown in Figure 5A — The speed of all movies is 40 times faster than the actual movements. [file mmc8.jpg]
